# Cortical Face-Selective Responses Emerge Early in Human Infancy

**DOI:** 10.1523/ENEURO.0117-24.2024

**Published:** 2024-07-16

**Authors:** Heather L. Kosakowski, Michael A. Cohen, Lyneé Herrera, Isabel Nichoson, Nancy Kanwisher, Rebecca Saxe

**Affiliations:** ^1^Department of Psychology, Center for Brain Science, Harvard University, Cambridge, Massachusetts 02138; ^2^Department of Brain and Cognitive Sciences, Massachusetts Institute of Technology, Cambridge, Massachusetts 02139; ^3^Department of Psychology and Program in Neuroscience, Amherst College, Amherst, Massachusetts 01002; ^4^Psychology Department, University of Denver, Denver, Colorado 80210; ^5^Tulane Brain Institute, Tulane University, New Orleans, Louisiana 70118

**Keywords:** cerebral cortex, faces, FFA, fMRI, infant brain, MPFC, OFA, STS

## Abstract

In human adults, multiple cortical regions respond robustly to faces, including the occipital face area (OFA) and fusiform face area (FFA), implicated in face perception, and the superior temporal sulcus (STS) and medial prefrontal cortex (MPFC), implicated in higher-level social functions. When in development, does face selectivity arise in each of these regions? Here, we combined two awake infant functional magnetic resonance imaging (fMRI) datasets to create a sample size twice the size of previous reports (*n* = 65 infants; 2.6–9.6 months). Infants watched movies of faces, bodies, objects, and scenes, while fMRI data were collected. Despite variable amounts of data from each infant, individual subject whole-brain activation maps revealed responses to faces compared to nonface visual categories in the approximate location of OFA, FFA, STS, and MPFC. To determine the strength and nature of face selectivity in these regions, we used cross-validated functional region of interest analyses. Across this larger sample size, face responses in OFA, FFA, STS, and MPFC were significantly greater than responses to bodies, objects, and scenes. Even the youngest infants (2–5 months) showed significantly face-selective responses in FFA, STS, and MPFC, but not OFA. These results demonstrate that face selectivity is present in multiple cortical regions within months of birth, providing powerful constraints on theories of cortical development.

## Significance Statement

Social cognition often begins with face perception. In adults, several cortical regions respond robustly to faces, yet little is known about when and how these regions first arise in development. To test whether face selectivity changes in the first year of life, we combined two datasets, doubling the sample size relative to previous reports. In the approximate location of the fusiform face area, superior temporal sulcus, and medial prefrontal cortex but not occipital face area, face selectivity was present in the youngest group. These findings demonstrate that face-selective responses are present across multiple lobes of the brain very early in life.

## Introduction

Faces are highly salient visual and social features of our environment. In human adults, many cortical regions show robust and selective responses to faces (Haxby et al., 2000; Dai and Scherf, 2023). We focus on four such regions: the occipital face area (OFA) in the inferior occipital gyrus (IOG; Gauthier et al., 2000), the fusiform face area (FFA) in the fusiform gyrus (Kanwisher et al., 1997), and regions in the superior temporal sulcus (STS) and the medial prefrontal cortex (MPFC). Here we ask when in development each of these regions first respond selectively to faces.

OFA and FFA are both visual regions, responding robustly to visually presented faces and much less to any other visual category. OFA is anatomically posterior to FFA and appears to encode face features or parts (Henriksson et al., 2015). FFA, on the other hand, appears to encode the presence and identity of a face more holistically (Grill-Spector et al., 2004; Yovel and Kanwisher, 2005). Individual neurons within FFA are highly face-selective (Axelrod et al., 2019; Khuvis et al., 2021), and electrically stimulating this area can distort or create face percepts (Parvizi et al., 2012; Rangarajan et al., 2014; Schalk et al., 2017; Jonas et al., 2018).

In contrast, STS and MPFC contain regions that respond robustly to faces, but these responses are modulated by social context, and the same regions also respond to socially relevant stimuli that are not faces. A region in STS is face-selective when compared to other visual non-face categories and preferentially responds to socially relevant movements of faces, like facial expressions and shifts of eye gaze (Pitcher et al., 2011), but also responds to human voices (Deen et al., 2020). Similarly, a region in MPFC is face-selective on visual tasks, but the response to faces is influenced by the faces’ social attributes such as moral goodness and attractiveness (LaBar, 2003; O’Doherty et al., 2003; Cheng et al., 2022). The same region in MPFC also responds to socially relevant diagrams and verbal narratives (Kosakowski et al., 2022b). In sum, in adults OFA, FFA, STS, and MPFC all have face-selective responses, though plausibly performing different visual and social functions.

Initial functional magnetic resonance imaging (fMRI) studies revealed substantial changes in the extent and magnitude of face-selective responses throughout childhood and early adolescence, suggesting that face selectivity is slow to develop (Golarai et al., 2007, 2010, 2015; Scherf et al., 2007; Peelen et al., 2009; Cohen Kadosh et al., 2011, 2013; Joseph et al., 2011; Haist et al., 2013; Natu et al., 2016; Nordt et al., 2021; Tian et al., 2021; Feng et al., 2022). On the other hand, human infants have at least modest preferential responses to faces in the approximate location of OFA, FFA, STS, and MPFC (Tzourio-Mazoyer et al., 2002; Lloyd-Fox et al., 2009, 2011, 2017; Ichikawa et al., 2010; Deen et al., 2017, Powell et al., 2018; Lisboa et al., 2020b). Most of these prior studies did not measure responses to other visual categories, to establish whether the responses were face-selective. One recent study reported face-selective responses in FFA in infants, but did not investigate STS or MPFC (Kosakowski et al., 2022a). Additionally, because of small sample sizes, prior studies could not resolve when face-selective responses in these regions first appear, within the first year of life. Thus, it remains an open question when each of these cortical regions first shows face-selective responses (Scott and Arcaro, 2023).

**Figure 1.**
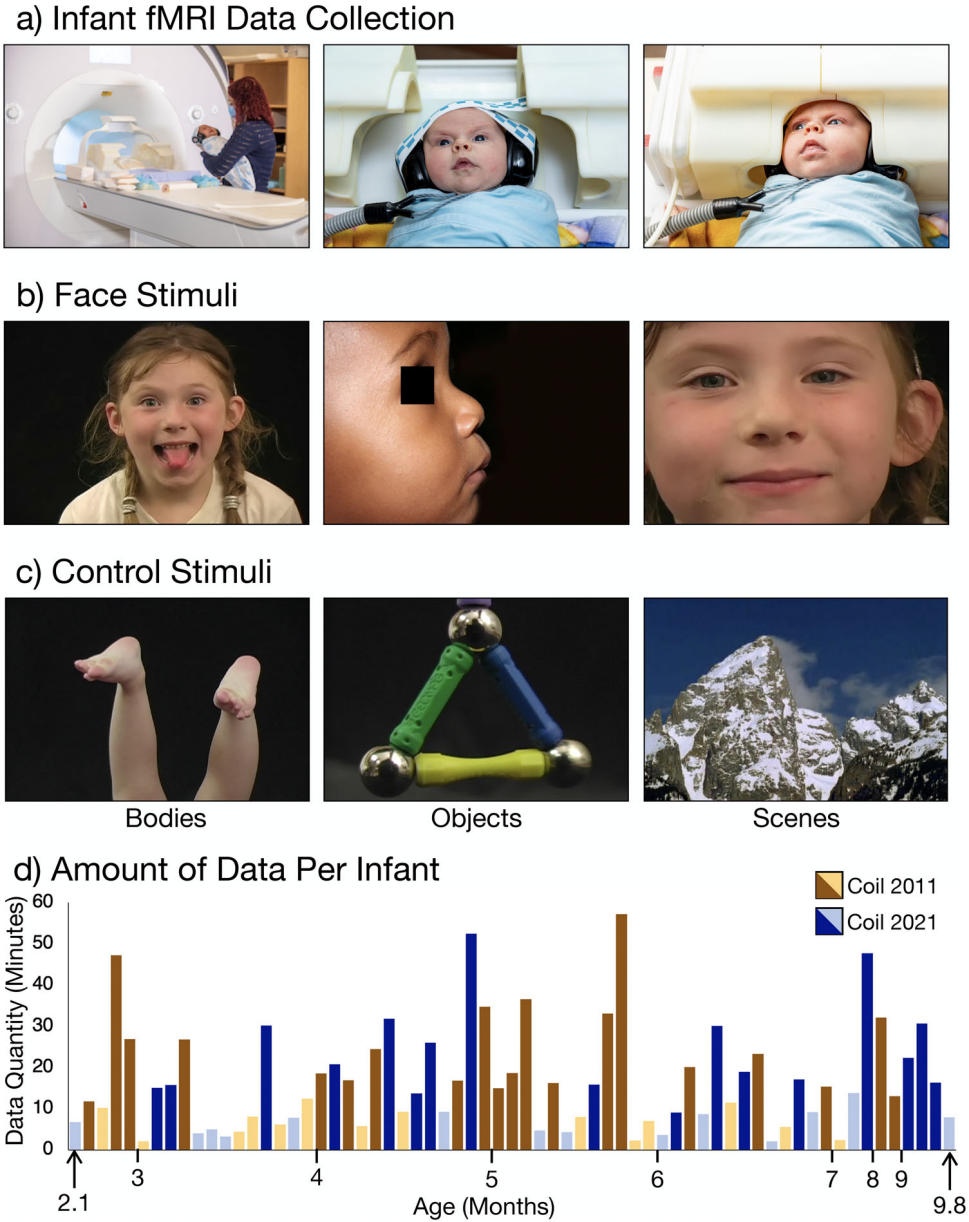
Awake infant fMRI scanning and stimuli. ***a***, For each MRI visit, we swaddled the infant, applied hearing protection, and placed the infant in a custom 32-channel infant head coil. Movies were projected in a mirror over infants’ eyes. Photo credit for images in the middle and right to Caitlin Cunningham Photography. Photo credit for the image on the left to Kris Brewer. ***b***, Example frames from dynamic face stimuli. ***c***, Example frames from dynamic body, object, and scene stimuli. ***d***, Infants that had enough usable data to compute a whole-brain contrast image are plotted on the *x*-axis, ordered by age in months. Data collected using Coil 2011 are indicated in brown; data collected using Coil 2021 are indicated in blue. The darker color indicates inclusion in fROI analyses (see Materials and Methods for inclusion criteria).

fMRI is the only neuroimaging method that has the coverage and spatial resolution to measure neural responses simultaneously in OFA, FFA, STS, and MPFC. A substantial challenge for fMRI with infant populations is that fMRI requires the participant to be still for long periods of time during data acquisition making awake infant fMRI studies rare (Ellis et al., 2020). For the current study, we combined two fMRI datasets that were collected (Fig. 1*a*), while infants watched dynamic videos of faces (Fig. 1*b*), bodies, objects, and scenes (Fig. 1*c*). In this combined dataset, infants ranged in age from 2 to 9 months (*n* = 65; Fig. 1*d*). As a result, the oldest infants had three times as much postbirth experience as the youngest ones. Thus, these data allow us to test whether, and when, cortical face selectivity emerges in OFA, FFA, STS, and MPFC in the first year of human infants’ lives.

## Materials and Methods

### Infant fMRI data

To investigate possible age-related changes in face selectivity in each region, we made two key changes to our analyses, compared with Kosakowski et al. (2022a). First, to get the largest possible sample of infants in each age group, we combined data that were collected using two different infant head coils and three different sequences, adjusting for differences in spatial resolution and distortions and temporal signal-to-noise ratio (tSNR) by using larger parcels created using the Glasser atlas (Glasser et al., 2016). Second, we used a contrast of faces greater than the response to all non-face conditions (Kosakowski et al., 2022a used face > objects). Other differences between the two analysis streams are noted below.

One group of infants (*n* = 31) was scanned from June 2016 to July 2019 with one coil (Coil 2011 described below; Keil et al., 2011) using a sinusoidal acquisition sequence (Zapp et al., 2012) on a 3 T Siemens Trio Scanner. A second group of infants (*n* = 56) was scanned from July 2019 to February 2020 with a different coil (Coil 2021 described below; Ghotra et al., 2021) using a higher-resolution acquisition sequence (see below, Data collection) on a 3 T Siemens Prisma Scanner. Overall, the tSNR is lower in Coil 2011 than Coil 2021 data. Data from the the two coils do not differ significantly in age (Coil 2011 mean, 5.04 months; Coil 2021 mean, 5.61 months; *t*_(57.07)_ = −1.23; *p* = 0.22; ci = −1.50 to 0.36) or motion (Coil 2011 mean, 0.20; Coil 2021 mean, 0.19; *t*_(62.41)_ = 0.41; *p* = 0.68; ci = 0.20–0.19).

Each infant was scheduled for up to eight visits. Any usable data that were collected within a 30 d window on the same coil with the same acquisition sequence were analyzed as a single session [see below, Data selection (subrun creation)]. In total, we had 74 sessions from 61 individuals (2.0–9.8 months; mean, 5.2 months). For each session, we collected 4.30–112.70 min of data (mean, 34.92 min; SD, 22.45 min). To be eligible for analysis, we identified segments of usable data with <2 mm/radians of frame-to-frame displacement, resulting in 65 usable sessions (2.1–9.8 months; mean, 5.3) from 53 unique individuals with 2.05–57.25 min of usable data per session (Fig. 1*d*; mean, 16.99; SD, 12.82). Of these, 49 sessions from 46 unique individuals had enough data to be included in whole-brain random effects analyses, and 37 sessions from 33 unique individuals met the inclusion criteria for functional region of interest (fROI) analyses (Fig. 1*d*).

### Participants

Infants (*n* = 86; 2.0–11.9 months; mean, 5.4 months; 41 females) were recruited from the metro area in and around Boston, MA through word of mouth, fliers, and social media. These data have been previously reported in Kosakowski et al. (2022a), mainly reporting data from Coil 2021 and measuring responses only in visually responsive category-selective regions. Information about which infants were included in the present analyses compared with those in Kosakowski et al. (2022a) are included in a table on OSF (https://osf.io/h7rbv/). Here, the data were reanalyzed with a focus on face-responsive regions across the cerebral cortex and combining data from both Coil 2021 (Ghotra et al., 2021) and Coil 2011 (Keil et al., 2011) to create a larger sample. Usable data [see below, Data selection (subrun creation)] were collected from 65 infants (2.6–11.9 months; 24 females; 31 from Coil 2021 and 34 from Coil 2011). Parents of participants were provided parking or reimbursed travel expenses. Participants received a small compensation for each visit and, when possible, printed images of their brain. Parents of participants provided informed consent, and all protocols were approved by the Institutional Review Board at MIT.

### Experimental paradigms

#### Paradigm 1

Infants watched videos of faces (Fig. 1*b*), bodies, objects, and scenes (Fig. 1*c*; Pitcher et al., 2011). A colorful, curvy, abstract baseline was used to maintain infants’ attention during baseline blocks. Videos were selected to be categorically homogeneous within blocks and heterogeneous between blocks. Each block was 18 s and was composed of six 3 s videos from the same category. Face videos showed one child's face on a black background. Object videos showed toys moving. Body videos showed children's hands or feet on a black background. Scene videos showed natural landscapes. Baseline blocks were also 18 s and consisted of six 3 s videos that featured abstract color scenes such as liquid bubbles or tie-dyed patterns. The block order was pseudorandom such that all blocks played once prior to playing again. Videos played continuously for as long as the infant was content, paying attention, and awake.

#### Paradigm 2

Infants watched videos from the same five conditions as in Paradigm 1. However, the videos were shortened to 2.7 s and interleaved with still images from the same category (but not drawn from the videos) presented for 300 ms. All blocks were 18 s and included six videos and six images. Video and image orders were randomized within blocks, and the block order was pseudorandom by category. Paradigm 2 contained one additional block depicting hand–object interactions which was not included in the present analysis.

### Data collection

Infants were swaddled if possible (Fig. 1*a*). A parent or researcher went into the scanner with the infant, while a second adult stood outside the bore of the scanner. Infants heard lullabies (https://store.jammyjams.net/products/pop-goes-lullaby-10) for the duration of the scan. For data collected with Coil 2011, lullabies were played over a loudspeaker into the scanning room. For data collected with Coil 2021, lullabies were played through custom infant headphones (Fig. 1*a*).

#### Coil 2011

For data collected with Coil 2011, we used a custom 32-channel infant coil designed for 3 T Siemens Trio Scanner (Keil et al., 2011) and a quiet EPI with sinusoidal trajectory (Zapp et al., 2012) with 22 near-axial slices [repetition time (TR), 3 s, echo time (TE), 43 ms; flip angle, 90°; field of view (FOV), 192 mm; matrix, 64 × 64; slice thickness, 3 mm; slice gap, 0.6 mm]. The sinusoidal acquisition sequence caused substantial distortions in the functional images.

#### Coil 2021

Infants wore custom infant MR-safe headphones. Infant headphones attenuated scanner noises and allowed infants to hear the lullabies. An adjustable coil design (Ghotra et al., 2021) increased infant comfort and accommodated headphones as well as a variety of head sizes (Fig. 1*a*). The new infant coil and infant headphones designed for 3 T Siemens Prisma Scanner enabled the use of an EPI with standard trajectory with 44 near-axial slices (TR, 3 s; TE, 30 ms; flip angle, 90°; FOV, 160 mm; matrix, 80 × 80; slice thickness, 2 mm; slice gap, 0 mm). Six infants had data collected using a different EPI with standard trajectory with 52 near-axial slices (TR, 2 s; TE, 30 ms; flip angle, 90°; FOV, 208 mm; matrix, 104 × 104; slice thickness, 2 mm; slice gap, 0 mm). Functional data collected with Coil 2021 were less distorted than data collected with Coil 2011.

### Data selection (subrun creation)

**Figure 2.**
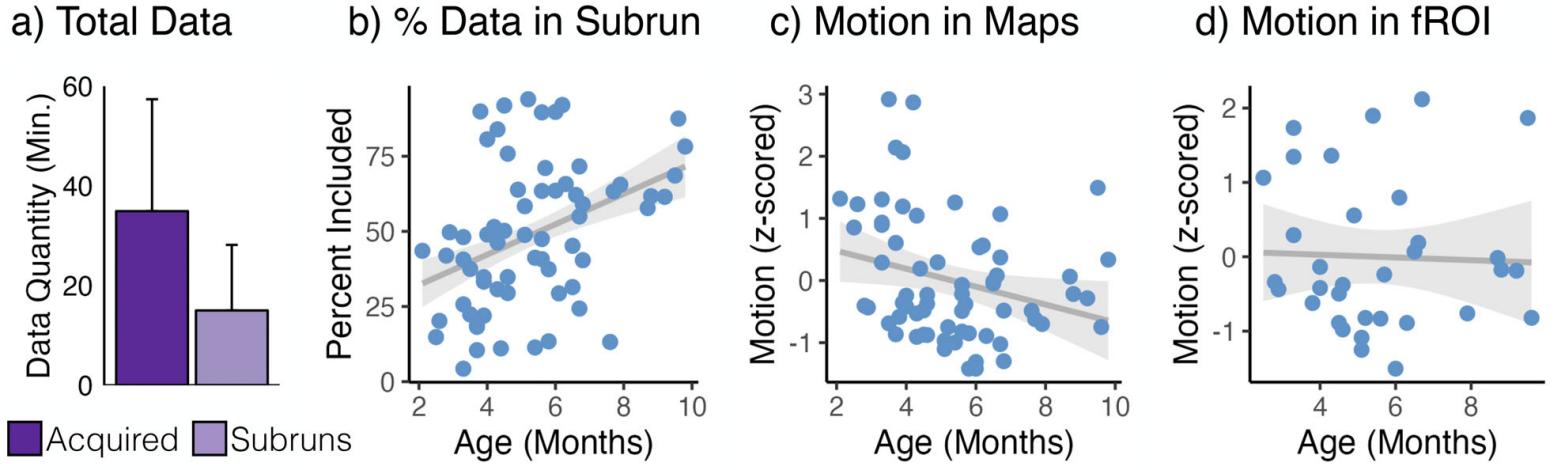
Data quality metrics for awake infant fMRI. ***a***, Variable amounts of data were acquired from each 30 day participant session (dark purple) and included in subruns (light purple). Bars indicate mean minutes of data per participant session; error bars are standard deviation. ***b***, The amount of data included in subruns for a participant session was correlated with age. ***c***, For participants that had enough data to compute whole-brain contrast maps, the proportion of motion (number of volumes with greater than 0.5 mm or radians of frame-to-frame displacement) was positively correlated with age. ***d***, For participants with enough data to be included in fROI analyses, motion was not correlated with age. Statistics supporting additional analyses of tSNR in Extended Data Figure 2-1.

To be included in the analysis, data had to meet criteria for low head motion (Deen et al., 2017; Kosakowski et al., 2022a). Data were cleaved between consecutive timepoints with >2 radians or millimeter of frame-to-frame displacement, creating subruns, which had to contain at least 24 consecutive low-motion volumes to be included in further analysis. All volumes included in a subrun were extracted from the original run data and combined to create a new NIfTI file for each subrun. Paradigm files indicating which condition occurred at each time point were similarly updated for each subrun. Volumes with greater than 0.5 radians or millimeter of frame-to-frame displacement from the previous or following volume were scrubbed (i.e., removed) from all analyses. Data collected within a 30 d window from a single subject were analyzed as one session. Figure 2*a* shows the amount of data collected and the amount of data included in the subruns, prior to scrubbing, for each participant session. The total amount of data initially collected was negatively correlated with age (*r* = −0.26; *p* = 0.003), and the proportion of data we retained per session was positively correlated with age (Fig. 2*b*; *r* = 0.39; *p* < 0.001). That is, older infants had shorter sessions and tended to be still for greater proportions of those sessions. Younger infants were more likely to move and required longer sessions to produce the same eventual yield of usable data.

10.1523/ENEURO.0117-24.2024.f2-1 Figure 2-1 **Analyses of temporal signal-to-noise ratio (tSNR) in awake infant fMRI data.** Plots show estimated effects of run length, run type (i.e., subruns (red) vs concatenated (blue)) on tSNR in (a) Coil 2011 and (b) Coil 2021 data. In Coil 2011 data (a), the tSNR decreases as a function of run length but the decrease is greater when the subruns are concatenated. In Coil 2021 data (b) the tSNR decreases as function of run length but this effect is not modulated by concatenating the runs. All estimated effects are plotted using plot_model from sjPlot package in R. Download Figure 2-1, TIF file.

Participants had to have at least 5 min of low-motion data to be included in whole-brain analyses. Only one session from each participant was included in each RFX analysis (which were run separately for Coil 2011 and Coil 2021). For fROI analyses, subruns were combined or split, as necessary, to create subruns with at least 96 volumes each. Subruns were designed to have approximately the same number of volumes, within participant (see below for more information). To be included in the fROI analysis, participants had to have at least two subruns (one to choose voxels and the other to extract independent response magnitudes from the selected voxels).

### fMRI data preprocessing

Each subrun was processed individually. First, an individual functional image was extracted from the middle of the subrun to be used for registering the subruns to one another for further analysis. Then, each subrun was motion corrected using FSL MCFLIRT. If >3 consecutive images had >0.5 mm or 0.5 radians of motion, there had to be at least seven consecutive low-motion volumes following the last high-motion volume for those volumes to be included in the analysis. Additionally, each subrun had to have at least 24 volumes after accounting for motion and timepoints when the infants appeared to be asleep (e.g., with eyes closed). Functional data were skull-stripped (FSL BET2), intensity normalized, and spatially smoothed with a 3 mm FWHM Gaussian kernel (FSL SUSAN).

### Data registration

All subruns were aligned within subjects, and then each subject was registered to a standard template. First, the middle image of each subrun was extracted and used as an example image for registration. If the middle image was corrupted by motion or distortion, a better image was selected as the example image. The example image from the middle subrun of the first visit with usable data was used as the target image. All other subruns from each subject were registered to that subject's target image using FSL FLIRT. The target image for each subject was then registered to a template image using FSL FLIRT. For data collected with Coil 2011, the template image was taken from Deen et al. (2017). For data collected with Coil 2021, the template image was taken from Kosakowski et al. (2022a). Given the distortion of the images and the lack of an anatomical image for each subject, traditional registration tools do not effectively register infant data between subjects. As such, we attempted to register each image using a rigid, an affine, and a partial affine registration with FSL FLIRT. The best image registration was selected by eye from the three options and manually tuned using the FreeSurfer GUI for the best possible data alignment. Each image took between 2 and 8 h of human labor to register. Images collected with Coil 2021 were transformed into the anatomical space of the template image for visualization.

A potential concern is the impact of concatenating subruns on the measured timecourses. To address this, we directly tested the effect of analytic partition types (i.e., subruns vs concatenated subruns) on the tSNR across all voxels in all parcels (see below, fROI analysis, for information about parcels). The model also included predictors for run length, coil, and parcel and only included runs with at least 96 volumes. There was not a main effect of partition type [i.e., subruns with vs without concatenation (*F*_(1/576.58)_ = 0.95; *p* = 0.33)], but tSNR was lower in longer runs (*F*_(2/82.27)_ = 26.42; *p* < 0.00001) and higher in data collected using Coil 2021 (*F*_(2/82.27)_ = 26.42; *p* < 0.00001), and there was a significant three-way interaction between run length, coil, and partition type (*F*_(2/567.55)_ = 3.49; *p* = 0.03). In Coil 2011 data (Extended Data Fig. 2-1*a*), tSNR decreased as a function of run length and was more pronounced in concatenated subruns (*F*_(1/332.42)_ = 10.44; *p* = 0.001), but there was no such effect in Coil 2021 data (Extended Data Fig. 2-1*b*; *F*_(1/288.20)_ = 0.0009; *p* = 0.98). These results demonstrate that Coil 2011 data have lower tSNR, likely due to differential distortion patterns.

### Subject-level beta and contrast maps

Functional data were analyzed with a whole-brain voxel–wise general linear model (GLM) using custom MATLAB scripts. The GLM included four condition regressors (faces, bodies, objects, and scenes), six motion regressors, a linear trend regressor, and five principal component analysis (PCA) noise regressors. PCA noise regressors are analogous to GLMdenoise (Kay et al., 2013). Condition regressors were defined as a boxcar function for the duration of each condition block (18 s). Infant inattention or sleep was accounted for using a single nuisance (“sleep”) regressor. The sleep regressor was defined as a boxcar function with a 1 for each TR the infant was not looking at the stimuli, and the corresponding TR was set to 0 for all condition regressors. Boxcar condition and sleep regressors were convolved with an infant hemodynamic response function (HRF) that is characterized by a longer time to peak and deeper undershoot compared with the standard adult HRF (Arichi et al., 2012). Next, data and all regressors except PCA noise regressors were concatenated across subruns. PCA noise regressors were computed across concatenated data, and beta values were computed for each condition in a whole-brain voxel–wise GLM. Subject-level contrast maps to test for face-selective responses were computed as the difference between the face beta and the average of all nonface (i.e., bodies, objects, and scenes) betas for each voxel using in-house MATLAB code.

### Group random effect analysis

Due to variable distortions in the BOLD images across participants, and lack of a T1 and/or T2 image from most infants, data registration across participants is imperfect. Additionally, the sequences used with each coil created very different patterns of spatial distortion, so we conducted separate group random effect analyses for data collected from each coil. First, subject-level contrast difference (faces–nonface) maps were transformed into coil-specific template space. Group RFX analyses were performed using FreeSurfer mri_concat and FreeSurfer mri_glmfit. In the dataset used for whole-brain analyses, the amount of motion (the number of scrubbed volumes divided by the number of total volumes) was negatively correlated with age (Fig. 2*c*; *r* = −0.27; *p* = 0.03).

### fROI analysis

Group RFX analyses are imperfect because they rely on high-quality registrations to a common template across subjects and they do not respect idiosyncratic anatomical and functional differences across individuals. Thus, to determine if cortical responses are face-selective, we utilized an fROI approach. Using fROI analyses enables us to (1) account for individual anatomical variability (Saxe et al., 2006), (2) more rigorously characterize responses using a cross-validation procedure (Nieto-Castañón and Fedorenko, 2012), and (3) require high-quality within–subject registrations while tolerating imperfect across-subject registrations.

We account for the variable amount of data in each subrun for each subject (*n* = 37 sessions; 33 unique individuals) and the impact this could have on reliable parameter estimates from the GLM by first combining or splitting subruns. This allowed us to approximately equate the amount of data across subruns within each subject. For example, if a subject had three subruns and the first had 30 volumes, the second had 75 volumes, and the third had 325 volumes, then we concatenated the first two subruns to create one subrun, and we split the third subrun into three resulting in a total of four subruns with approximately 100 volumes each. For data included in fROI analyses, motion and age were not correlated (Fig. 2*d*; *r* = −0.04; *p* = 0.8). Thus, the fROI analyses allow for a measurement of age effects that is not confounded with motion.

To constrain search areas for voxel selection, we used anatomically defined parcels transformed to subject-specific BOLD space. Due to the distortions in the Coil 2011 dataset, we opted to use larger parcels than the FFA and OFA parcels used in Kosakowski et al. (2022a). We created large parcels that extended well beyond the boundaries of face regions in the IOG (the approximate locations of OFA) and ventral temporal cortex (VTC, the approximate location of FFA), STS, and MPFC using the Glasser atlas (Glasser et al., 2016). The large OFA parcel included Glasser areas LO1, LO2, LO3, V4, V4t, and PIT. The large FFA parcel included Glasser areas VMV1, VMV2, VMV3, VVC, PHA1, PHA2, PHA3, and FFC. For the MPFC parcel, we used Glasser areas p24, d32, 9m, and p32. For the STS parcel, we used Glasser areas STSvp, STSva, STSdp, STSda, and STV. All parcels were transformed into infant-specific functional space by concatenating the subrun-to-infant template registration matrix with the infant template-to-MNI registration matrix and inverting those transformations.

We used an iterative leave-one-subrun-out procedure such that data were concatenated across all subruns except one. Then, whole-brain voxel–wise GLMs and contrast maps were computed. The top 5% of voxels that had a greater response to faces than the average response to nonface conditions within an anatomical constraint parcel were selected as the fROI for that subject. Then, the parameter estimates (i.e., beta values) for all four conditions were extracted from the left-out subrun. For all bar plots, beta values were averaged across participant sessions.

To determine whether a region's response was category-selective, we fit the beta values using a linear mixed effects model. In each model, we indicator-coded the three control conditions to test the hypothesis that the response to each control condition was significantly lower than the response to the face condition. Specifically, we fit a model in R using the lme4 software package (Bates et al., 2014) with the following expression:

lmer(betas∼bod+obj+scn+motion+age+coilID+(1|subject)).

There were three indicator-coded regressors to indicate which condition the beta was from. The “bod” regressors had 1 if the beta indicated a response to bodies and 0 for betas that were from other conditions. Similarly, the “obj” regressor had 1 for object betas and 0 for all other conditions, and the “scn” regressor had 1 for all scene betas and 0 for all other conditions. Motion was computed as the proportion of scrubbed volumes; coilID indicated if the data were collected on Coil 2011 or Coil 2021. Age and motion were *z*-scored. Motion and coil were fixed effects parameters of no interest, and the subject was coded as a random effect for all models. Four individual infants contributed more than one session of data, which was treated as within-subject variance through the random effect and the age regressor. The response in a parcel was deemed selective if the fixed effect coefficient for each of the three control conditions (i.e., bod, obj, and scn) was significantly negative. Because predictions were unidirectional, reported *p* values are one-tailed.

To test for condition by age interactions, we used the following model in R:

anova(lmer(betas∼condName*age+motion+coilID+(1|subject))),

where condName was the condition label for each beta value and age and motion were *z*-scored. Post hoc analyses of the effect of age on each condition were tested with the following model in R:

lmer(condBeta∼age+motion+coilID+(1|subjID)),

where condBeta was a vector with beta values from a single condition and age was *z*-scored. In instances where the model was singular due to negligible variance from the subjID random effect, we additionally fit a linear model, [lm(condBeta ∼ age + motion + coilID)], to ensure we obtained the same results.

To test for laterality effects between the left and right hemispheres, we fit the following model for each fROI in R:

anova(lmer(betas∼condName*hemi*age+motion+coilID+(1|subject))),

where condName was the condition label for each beta, the hemisphere indicated the left or right hemisphere for each beta, motion and age were *z*-scored, and the subject was included as a random effect. We tested for effects of laterality in the approximate locations of OFA, FFA, and STS, but not MPFC because the MPFC fROI is on the midline and the two hemispheres could not be reliably distinguished. For post hoc analyses of the effect of the hemisphere on each condition, we fit the following model in R for IOG, VTC, and STS:

anova(lmer(beta∼age*hemisphere+coilID+(1|subject))),

where beta was a vector of beta values from a single condition and the hemisphere indicated whether the beta value was from the left or right hemisphere. Age was *z*-scored, and the subject was included as a random effect.

To test if EVC has a different functional profile than face-selective regions, we fit the following model in R:

anova(lmer(betas∼condName*fROI+age+motion+coilID+(1|subject))),

where condName was the condition label for each beta (i.e., face, body, object, scene) and fROI indicated the region label for each beta (i.e., EVC and IOG or VTC or STS or MPFC). Motion and age were *z*-scored. We ran four models to test for a significant fROI by condition interaction between EVC and each face-selective fROI.

We also computed weighted LME models to account for the variable amount of data each subject contributed to each condition. The results were similar for these additional models and are reported in Extended Data Tables S1–S3.

### Code and data availability

All code is provided in a repository on Open Science Framework (OSF; https://osf.io/h7rbv/). All data for the fROI analysis, figures with face activations for all participants, and infant ID comparison to Kosakowski et al. (2022a) is also provided on OSF.

## Results

### Whole-brain contrast maps

**Figure 3.**
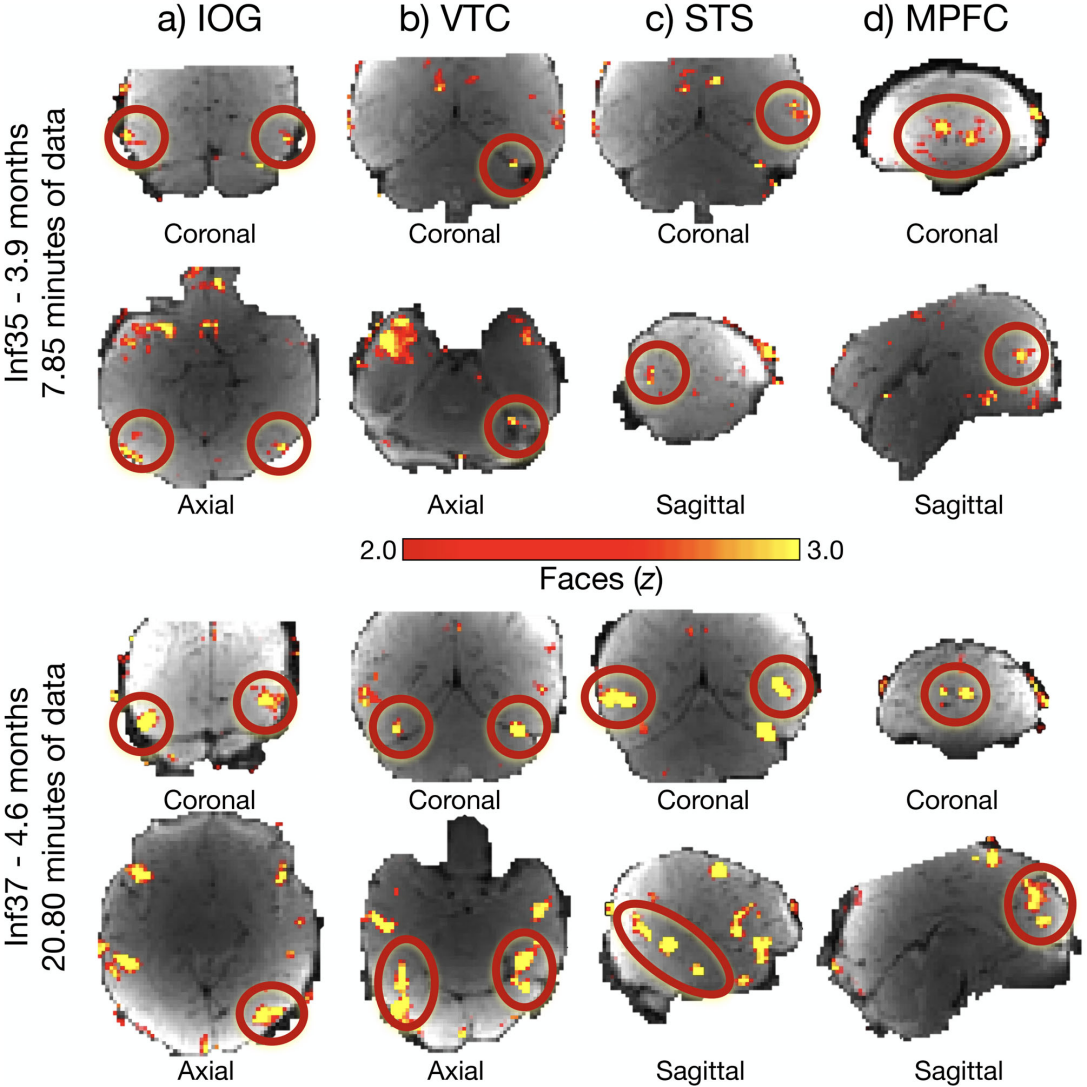
Cortical responses to faces in individual infants. Similarly aged individual infants with variable amounts of data (top panel, Inf35, 3.9 months, 7.85 min of data; bottom panel, Inf37, 4.6 months, 20.80 min of data) have face activations (faces > nonfaces) in IOG (***a***), the approximate location of OFA in adults; VTC (***b***), the approximate location of FFA in adults; STS (***c***); and MPFC (***d***). Relevant activations for each region are circled in red. Whole-brain contrast maps (faces > nonfaces) are displayed on a template BOLD image, truncated, and thresholded at *z* = 2.0–3.0 to enable visualization of face activations and allow for a direct comparison of activations between the two infants. Slice view is indicated below each image; the left hemisphere is on the left. Activations for every infant are available on OSF (https://osf.io/h7rbv/). Information aligning infants to Kosakowski et al. (2022a) is available on OSF (https://osf.io/h7rbv/).

First, we asked if infants have face responses in the approximate locations of OFA, FFA, STS, and MPFC by visualizing whole-brain maps in individual infants. At a lenient statistical threshold (*p* < 0.01, uncorrected), visual inspection of contrast maps (faces > nonfaces) from all sessions with sufficient usable data (*n* = 65) revealed face activations in the approximate location of OFA, FFA, STS, and MPFC in many infants scanned on both Coil 2011 and Coil 2021. Importantly, infants with different amounts of data had face responses in the approximate location of each expected region (e.g., Fig. 3; all infants on OSF; https://osf.io/h7rbv/).

**Figure 4.**
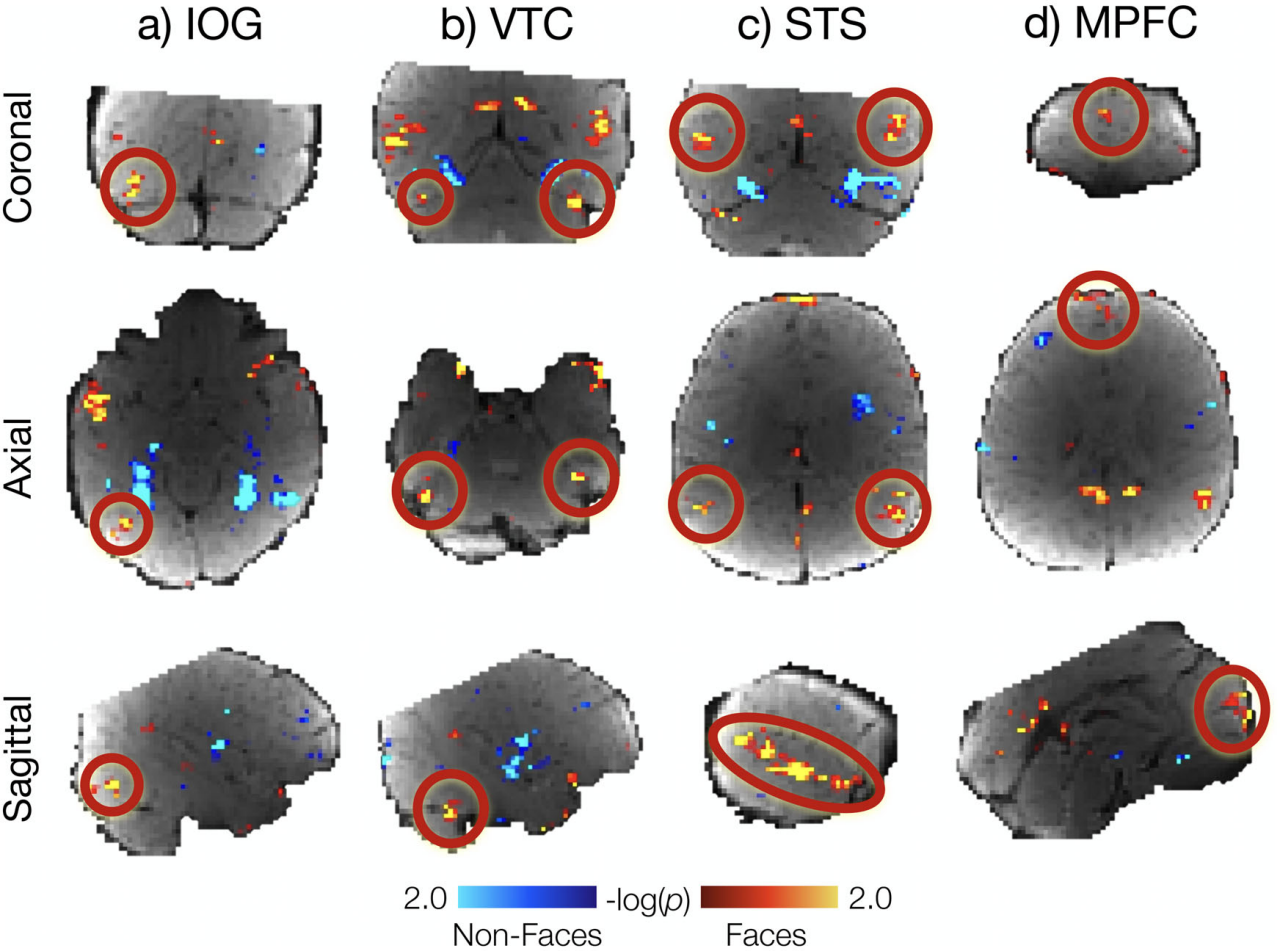
Group face responses in the infant cerebral cortex. Whole-brain group random effect analysis of Coil 2021 (*n* = 23) at a lenient threshold (*p* < 0.05) revealed face activations (faces > nonfaces) in (***a***) IOG, the approximate location of OFA in adults; (***b***) VTC, the approximate location of FFA in adults; (***c***) STS; and (***d***) MPFC. Hot colors indicate face activations; cool colors indicate average response to nonfaces. Activation clusters did not survive correction for multiple comparisons. Activations for each region are shown on infant template BOLD image in coronal (top row), axial (middle row), and sagittal (bottom row) views and highlighted with a red circle. Results for Coil 2011 data are visualized in Extended Data Figure 4-1.

The Coil 2021 group RFX map (Fig. 4) showed face > nonface activations in the approximate locations of OFA, FFA, STS, and MPFC. For Coil 2011 group RFX, there were face > nonface activations in the approximate locations of STS and MPFC but not in the approximate locations of OFA or VTC (Extended Data Fig. 4-1). These activations did not survive correction for multiple comparisons, though note that registration across infants was only approximate, given the highly distorted functional images and the absence of a high-resolution individual anatomical image for most individuals.

10.1523/ENEURO.0117-24.2024.f4-1 Figure 4-1 **Group face responses in infant cerebral cortex.** Whole brain group random effects analysis of Coil 2011 at a lenient threshold (*p *< 0.05) revealed face activations (faces > non-faces) in (c) superior temporal sulcus (STS), and (d) medial prefrontal cortex (MPFC). Face activations were not observed in the group in (a) inferior occipital gyrus (IOG), the approximate location of OFA in adults or (b) ventral temporal cortex (VTC), the approximate location of FFA in adults. Hot colors indicate face activations, cool colors indicate average response to non-faces. Activation clusters did not survive correction for multiple comparisons. Activations for each region are shown on infant template BOLD image in coronal (top row), axial (middle row) and sagittal (bottom row) views and highlighted with a red circle. Results for Coil 2021 data are visualized in Figure 4. Download Figure 4-1, TIF file.

### fROIs

**Figure 5.**
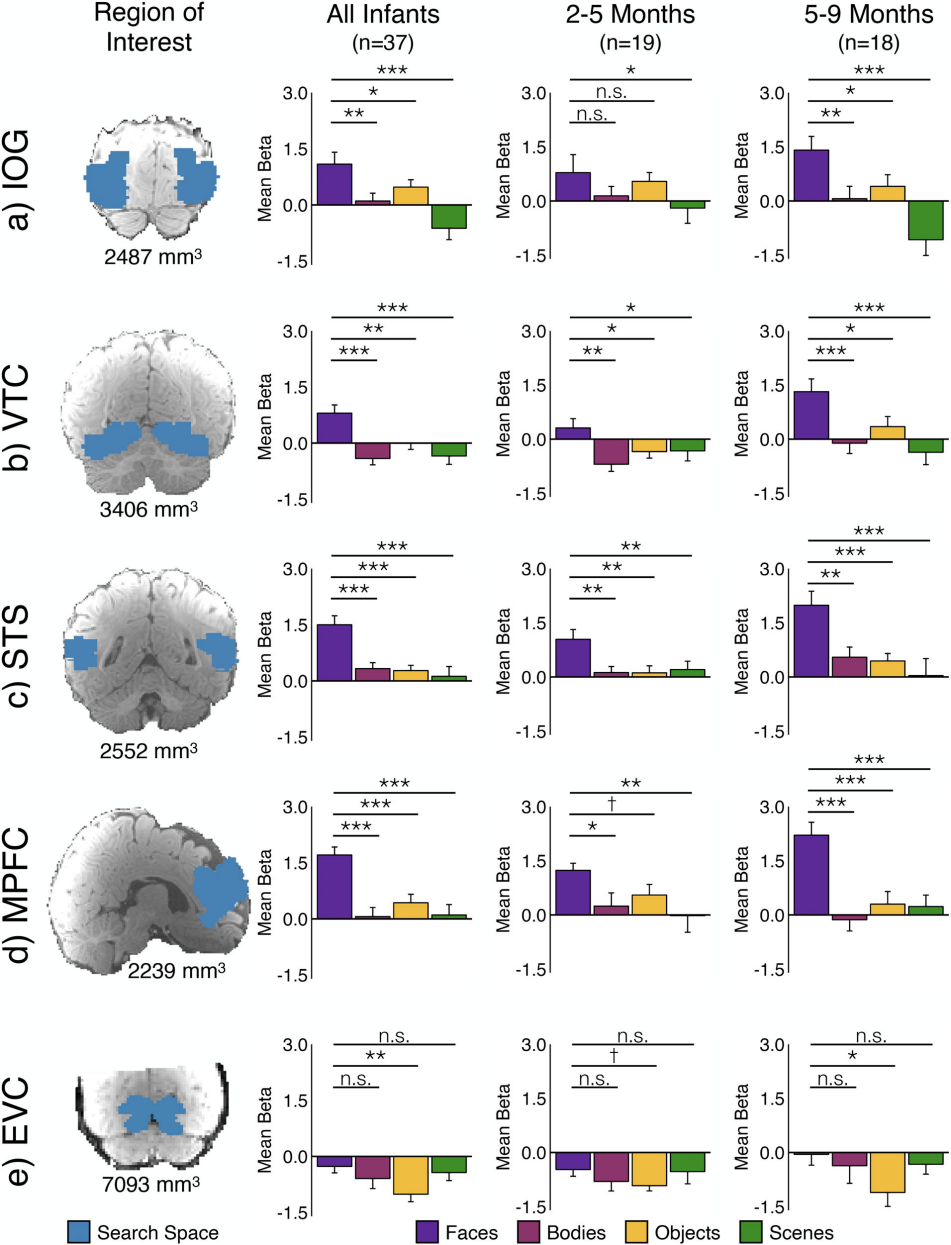
Face-selective responses in the infant cortex. In analyses collapsed across Coil 2011 and Coil 2021 datasets, we identified an fROI in each participant as the top 5% of voxels that responded more to faces than nonfaces within a large anatomical search space (first column, blue, projected onto an infant anatomical image) of (***a***) IOG, the approximate location of OFA; (***b***) VTC, the approximate location of FFA; (***c***) STS; (***d***) MPFC; and (***e***) EVC. For each region, cross-validated fROI analyses were conducted in all infants (*n* = 37) and separately in younger (*n* = 19) and older (*n* = 18) infants. Bar charts show the mean response across participants in each fROI to faces (purple), bodies (pink), objects (yellow), and scenes (green). Error bars indicate within-subject standard error (Cousineau, 2005). Symbols indicate one-tailed statistics from linear mixed effect models: ^†^*p* < 0.1; *p < 0.05; **p < 0.01; ***p < 0.001. Additional statistics reported in Tables 1 and 2; post hoc analyses of the response to each condition as a function age are reported in Extended Data Figure 5-1 and Table 3.

**Figure 6.**
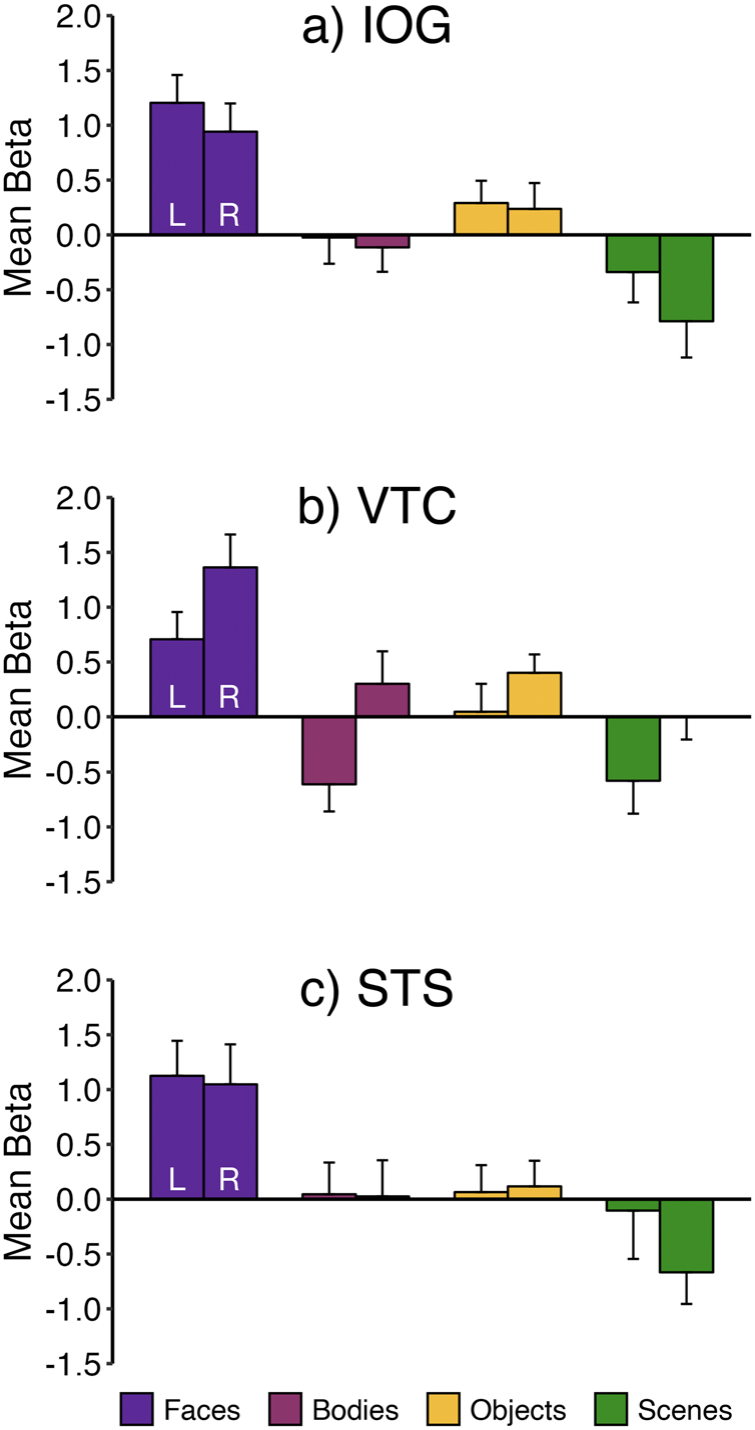
Face selectivity is bilateral. In analyses collapsed across Coil 2011 and Coil 2021 datasets, we identified an fROI for each participant in each hemisphere in (***a***) IOG, the approximate location of OFA; (***b***) VTC, the approximate location of FFA; and (***c***) STS. For each region, cross-validated fROI analyses were conducted in all infants (*n* = 37). Bar charts show the mean response across participants in each fROI to faces (purple), bodies (pink), objects (yellow), and scenes (green). Error bars indicate within-subject standard error (Cousineau, 2005). Additional visualization reported in Extended Data Figure 6-1 and statistics reported in Table 4, and Extended Data Tables 4-1–4-5.

All fROI analyses were conducted in *n* = 37 sessions (33 unique individuals) who had at least two subruns. IOG is the approximate location of OFA in adults. Across all infants in the IOG fROI analysis (Fig. 5*a*; Table 1), the response to faces was significantly greater than the response to bodies (*p* = 0.004), objects (*p* = 0.049), and scenes (*p* < 0.001). The overall magnitude of response across all conditions increased with age (*p* = 0.000006), and there was an age by condition interaction (*p* = 0.003; Table 2). Post hoc analyses revealed that the age by condition interaction was driven by a lower response to scenes in older infants (*p* = 0.003; Extended Data Fig. 5-1*a*; Table 3). Using a median split by age, we ran separate fROI analyses of younger and older infants (Fig. 5*a*; Table 1). In the younger infants alone, the response to faces was greater than the scene response (*p* = 0.03) but not significantly different from the object or body responses (*p*s > 0.1), but in the older infants alone, the face response was significantly greater than the response to each other condition (all *p*s < 0.03). To test for any laterality effects, we analyzed left and right hemispheres separately. Despite a hemisphere by age interaction (*p* = 0.04; Table 4) and a condition by age interaction (*p* = 0.03), the condition by hemisphere by age interaction was not significant (*p* > 0.3). Post hoc analyses indicated there was no difference in responses to faces in right versus left hemispheres (*p* > 0.4; Fig. 6*a*; Extended Data Table 4-4).

10.1523/ENEURO.0117-24.2024.f5-1 Figure 5-1 **Effect of age on the response magnitude for each condition in each fROI.** Scatter plots show magnitudes for each condition from fROI analyses collapsed across Coil 2011 and Coil 2021 datasets as a function of age. ROIs include (a) inferior occipital gyrus (IOG), the approximate location of OFA, (b) ventral temporal cortex (VTC), the approximate location of FFA, (c) superior temporal sulcus (STS), (d) medial prefrontal cortex (MPFC), and (e) early visual cortex (EVC). Face betas are plotted in purple, body betas are plotted in pink, object betas are plots in yellow, and scene betas are plotted in green. Age is z-scored. Symbols indicate statistics from linear mixed effects models: *p *< 0.05. Additional statistics reported in Table 3. Download Figure 5-1, TIF file.

10.1523/ENEURO.0117-24.2024.f6-1 Figure 6-1 **Effect of age on the response magnitude for each condition in each fROI.** Scatter plots show magnitudes for each condition from fROI analyses collapsed across Coil 2011 and Coil 2021 datasets as a function of age. ROIs include (a, b) left and right inferior occipital gyrus (IOG), the approximate location of OFA, (c, d) left and right ventral temporal cortex (VTC), the approximate location of FFA, and (e, f) superior temporal sulcus (STS). Age is z-scored. Face betas are plotted in purple, body betas are plotted in pink, object betas are plots in yellow, and scene betas are plotted in green. Symbols indicate statistics from linear mixed effects models: *p *< 0.1; *p *< 0.05; **p < 0.01. Additional statistics reported in Table 4-2. Download Figure 6-1, TIF file.

**Table 1.** Face selectivity in fROIs

fROI	Intercept	Bodies	Objects	Scenes	Age	Motion	Coil
All infants							
IOG	**1.08 (0.50)**	**−1.07 (0.40)**	**−0.67 (0.40)**	**−2.05 (0.40)**	−0.39 (0.24)	**−0.59 (0.24)**	0.14 (0.58)
VTC	**0.53 (0.26)**	**−1.19 (0.29)**	**−0.71 (0.29)**	**−1.21 (0.29)**	−0.17 (0.12)	**0.32 (0.12)**	*0.47 (0.26)*
STS	**1.67 (0.28)**	**−1.08 (0.25)**	**−1.17 (0.25)**	**−1.03 (0.25)**	0.12 (0.14)	0.05 (0.14)	*−0.56 (0.32)*
MPFC	**1.91 (0.40)**	**−1.63 (0.33)**	**−1.30 (0.33)**	**−1.50 (0.33)**	−0.03 (0.20)	0.07 (0.20)	−0.48 (0.46)
EVC	*−0.58 (0.37)*	−0.35 (0.32)	**−0.82 (0.32)**	−0.20 (0.32)	*−0.30 (0.18)*	0.17 (0.18)	0.63 (0.42)
Youngest infants							
IOG	0.20 (0.82)	−0.64 (0.52)	−0.24 (0.52)	**−0.98 (0.52)**	−0.29 (0.52)	0.52 (0.52)	1.02 (0.99)
VTC	−0.08 (0.36)	**−1.01 (0.33)**	**−0.66 (0.33)**	**−0.64 (0.33)**	*−0.38 (0.20)*	0.21 (0.20)	*0.67 (0.39)*
STS	**1.25 (0.42)**	**−0.92 (0.31)**	**−0.93 (0.31)**	**−0.84 (0.31)**	0.21 (0.26)	−0.35 (0.26)	−0.36 (0.49)
MPFC	**1.20 (0.70)**	**−0.99 (0.49)**	*−0.68 (0.49)*	**−1.25 (0.49)**	0.10 (0.43)	−0.24 (0.43)	0.05 (0.83)
EVC	**−0.96 (0.57)**	−0.33 (0.35)	*−0.45 (0.35)*	−0.06 (0.35)	−0.41 (0.36)	0.57 (0.36)	0.84 (0.69)
Oldest infants							
IOG	**2.25 (0.51)**	**−1.53 (0.59)**	**−1.11 (0.59)**	**−3.18 (0.59)**	−0.27 (0.26)	**−0.71 (0.26)**	**−1.10 (0.52)**
VTC	**1.24 (0.39)**	**−1.39 (0.44)**	**−0.77 (0.44)**	**−1.81 (0.44)**	0.01 (0.19)	−0.00 (0.19)	*0.10 (0.39)*
STS	**1.89 (0.38)**	**−1.25 (0.37)**	**−1.43 (0.37)**	**−1.24 (0.37)**	*0.38 (0.21)*	0.17 (0.20)	−0.33 (0.42)
MPFC	**2.49 (0.49)**	**−2.31 (0.44)**	**−1.95 (0.44)**	**−1.77 (0.44)**	0.09 (0.29)	0.26 (0.27)	−0.64 (0.57)
EVC	−0.02 (0.47)	−0.37 (0.56)	**−1.21 (0.56)**	−0.34 (0.56)	−0.16 (0.22)	0.16 (0.23)	0.02 (0.46)

Parameter estimates from linear mixed effects models with beta values for each condition as predictors. Indicator-coded vectors used to test if body, object, and scene responses are each significantly less than the response to faces. Sex and *z*-scored age were coded as fixed effects, and the subject was coded as a random effect. Standard error is indicated in parentheses; *p* < 0.05 is indicated in bold; *p* < 0.10 is indicated in italics. A negative number in bold indicates a significantly lower response to that condition than to faces. The intercept indicates the magnitude of the face response relative to the baseline condition. Statistical models with weights added to account for variable amounts of data from each infant and each condition are reported in Extended Data Table 1-1.

10.1523/ENEURO.0117-24.2024.t1-1 Table 1-1 **Face selectivity in functional regions of interest with condition weights.** Parameter estimates from linear mixed effects models with beta values for each condition as predictors. Indicator-coded vectors used to test if body, object, and scene responses are each significantly less than the response to faces. Sex and z-scored age were coded as fixed effects and subject was coded as a random effect. Standard error is indicated in paratheses; *p *< 0.05 is indicated in bold; *p *< 0.10 is indicated in italics. A negative number in bold indicates a significantly lower response to that condition to faces. The intercept indicates the magnitude of the face response relative to baseline. Statistical models without weights are reported in Table 1. Download Table 1-1, DOC file.

**Table 2.** Interaction effects of age and fROI

Variable	Sum Sq.	Num. DF	Den. DF	*F*	*p*
IOG					
Condition	**81.99**	**3**	**103.42**	**10.14**	**0.000006**
*Z*-Scored age	**18.41**	**1**	**90.74**	**6.83**	**0.01**
*Z*-Scored motion	*7.51*	*1*	*89.15*	*2.79*	*0.10*
Coil	0.19	1	42.22	0.07	0.79
Condition * age	**40.96**	**3**	**103.42**	**5.07**	**0.003**
VTC					
Condition	**35.87**	**3**	**103.43**	**8.05**	**0.00007**
*Z*-Scored age	**9.75**	**1**	**50.01**	**6.57**	**0.01**
*Z*-Scored motion	2.79	1	49.24	1.88	0.18
Coil	*5.06*	*1*	*32.59*	*3.40*	*0.07*
Condition * age	*9.89*	*3*	*103.43*	*2.22*	*0.09*
STS					
Condition	**33.72**	**3**	**96.30**	**10.20**	**0.000007**
*Z*-Scored age	0.13	1	67.94	0.11	0.74
*Z*-Scored motion	0.67	1	66.92	0.61	0.44
Coil	*3.42*	*1*	*31.70*	*3.11*	*0.09*
Condition * age	6.01	3	96.30	1.82	0.15
MPFC					
Condition	**62.72**	**3**	**107.72**	**10.32**	**0.000005**
*Z*-Scored age	0.28	1	87.36	0.14	0.71
*Z*-Scored motion	0.05	1	86.22	0.03	0.87
Coil	2.24	1	44.74	1.11	0.30
Condition * age	7.35	3	107.72	1.21	0.31
EVC					
Condition	*13.47*	*3*	*105.34*	*2.41*	*0.07*
*Z*-Scored age	1.52	1	78.69	0.82	0.37
*Z*-Scored motion	*5.24*	*1*	*77.72*	*2.81*	*0.10*
Coil	4.35	1	40.39	2.33	0.13
Condition * age	11.38	3	105.34	2.03	0.11
IOG and VTC					
fROI	5.46	1	245.21	2.30	0.13
Condition	**106.50**	**3**	**245.21**	**14.97**	**0.000000006**
*Z*-Scored age	1.08	1	100.53	0.46	0.50
*Z*-Scored motion	3.96	1	98.40	1.67	0.20
Coil	2.53	1	43.70	1.07	0.31
fROI * condition	11.36	3	245.21	1.60	0.19
fROI * age	**12.01**	**1**	**245.21**	**5.06**	**0.03**
Condition * age	**44.74**	**3**	**245.21**	**6.29**	**0.0004**
fROI * condition * age	6.12	3	245.21	0.86	0.46
EVC and IOG					
fROI	**56.10**	**1**	**244.38**	**24.15**	**0.000002**
Condition	**48.86**	**3**	**244.38**	**7.01**	**0.0002**
*Z*-Scored age	*8.15*	*1*	*134.70*	*3.51*	*0.06*
*Z*-Scored motion	**9.52**	**1**	**130.07**	**4.10**	**0.04**
Coil	2.77	1	51.54	1.19	0.28
fROI * condition	**46.60**	**3**	**244.38**	**6.69**	**0.0002**
fROI * age	**11.88**	**1**	**244.38**	**5.11**	**0.02**
Condition * age	**39.81**	**3**	**244.38**	**5.71**	**0.0009**
fROI * condition * age	12.52	3	244.38	1.80	0.15
EVC and VTC					
fROI	**26.55**	**1**	**244.74**	**15.09**	**0.0001**
Condition	**31.16**	**3**	**244.74**	**5.90**	**0.0007**
*Z*-scored age	**7.78**	**1**	**86.78**	**4.42**	**0.04**
*Z*-Scored motion	3.14	1	85.36	1.79	0.19
Coil	**7.82**	**1**	**40.51**	**4.44**	**0.04**
fROI * condition	**18.17**	**3**	**244.74**	**3.44**	**0.02**
fROI * age	0.0004	1	244.74	0.0002	0.99
Condition * age	**14.81**	**3**	**244.74**	**2.81**	**0.04**
fROI * condition * age	6.46	3	244.74	1.22	0.30
EVC and STS					
fROI	**95.36**	**1**	**244.35**	**43.57**	**0.0000000003**
Condition	**39.10**	**3**	**244.35**	**5.96**	**0.0006**
*Z*-Scored age	4.18	1	61.29	1.91	0.17
*Z*-Scored motion	0.36	1	60.60	0.17	0.68
Coil	0.01	1	35.36	0.00	0.96
fROI * condition	8.09	3	244.35	1.23	0.30
fROI * age	0.65	1	244.35	0.30	0.59
Condition * age	*15.01*	*3*	*244.35*	*2.29*	*0.08*
fROI * condition * age	2.38	3	244.35	0.36	0.78
EVC and MPFC					
fROI	**105.05**	**1**	**247.45**	**37.46**	**0.000000004**
Condition	**53.49**	**3**	**247.45**	**6.36**	**0.0004**
Z-Scored age	2.78	1	74.04	0.99	0.32
Z-Scored motion	3.48	1	73.22	1.24	0.27
Coil	0.27	1	40.29	0.10	0.76
fROI * condition	**22.70**	**3**	**247.45**	**2.70**	**0.046**
fROI * age	1.47	1	247.45	0.52	0.47
Condition * age	10.83	3	247.45	1.29	0.28
fROI * condition * age	7.89	3	247.45	0.94	0.42

All results from linear mixed effect models are converted to ANOVA table with R function anova; *p* < 0.05 is indicated in bold; *p* < 0.10 is indicated in italics. The same models with weights added to account for variable amount of data collected from each infant and each condition are in Extended Data Table 2-1. Models testing for interaction between age and condition in each hemisphere are in Extended Data Table 2-2 (without weights) and Extended Data Table 2-3 (with weights).

10.1523/ENEURO.0117-24.2024.t2-1 Table 2-1 **Interaction effects of age and fROI with condition weights.** All results from linear mixed effects models converted to ANOVA table with R function anova; *p *< 0.05 is indicated in bold, *p *< 0.10 is indicated in italics. The same models without weights reported in Table 2. Models testing for interaction between age and condition are in each hemisphere are in Table 2-2 (without weights) and Table 2-3 (with weights). Download Table 2-1, DOC file.

10.1523/ENEURO.0117-24.2024.t2-2 Table 2-2 **Interaction effects of age in each fROI for each hemisphere.** All results from linear mixed effects models converted to ANOVA table with R function anova; *p *< 0.05 is indicated in bold, *p *< 0.10 is indicated in italics. Models with weights are in Table 4-4. Download Table 2-2, DOC file.

10.1523/ENEURO.0117-24.2024.t2-3 Table 2-3 **Interaction effects of age in each fROI for each hemisphere with condition weights.** All results from linear mixed effects models converted to ANOVA with R function anova; *p *< 0.05 is indicated in bold, *p *< 0.10 is indicated in italics. Models without weights are in Table 4-3. Download Table 2-3, DOC file.

**Table 3.** Effect of age on each condition

fROI	Intercept^a^	Age^a^	Motion^a^	Coil^a^
IOG face^b^	**1.72 (0.72)**	0.40 (0.49)	*−0.84* *(0.49)*	−0.92 (0.99)
IOG body^b^	0.12 (0.50)	0.03 (0.34)	−0.37 (0.34)	0.06 (0.69)
IOG object^b^	0.39 (0.48)	−0.13 (0.33)	−0.01 (0.33)	0.31 (0.66)
IOG scene	**−1.21 (0.44)**	**−0.99 (0.29)**	−0.48 (0.29)	0.65 (0.60)
VTC face^b^	**0.99 (0.34)**	**0.49 (0.24)**	−0.29 (0.23)	−0.38 (0.47)
VTC body^b^	**−0.66 (0.29)**	0.33 (0.20)	**−0.47 (0.19)**	0.46 (0.39)
VTC object^b^	−0.44 (0.29)	**0.45 (0.20)**	−0.18 (0.20)	**0.95 (0.40)**
VTC scene	**−0.82 (0.34)**	−0.08 (0.23)	0.01 (0.23)	0.73 (0.46)
STS face^b^	**2.00 (0.38)**	0.33 (0.26)	0.36 (0.26)	**−1.18 (0.52)**
STS body^b^	0.37 (0.34)	0.38 (0.23)	**0.51 (0.23)**	−0.15 (0.47)
STS object	0.25 (0.28)	0.12 (0.19)	0.20 (0.19)	−0.11 (0.38)
STS scene^b^	*0.47* *(0.26)*	−0.10 (0.18)	**0.42 (0.17)**	−0.27 (0.35)
MPFC face	**2.24 (0.45)**	0.06 (0.15)	*−0.74* *(0.15)*	−1.24 (0.47)
MPFC body	−0.22 (0.53)	−0.19 (0.34)	0.18 (0.34)	0.36 (0.72)
MPFC object	*0.88* *(0.46)*	0.02 (0.29)	0.32 (0.29)	−0.85 (0.62)
MPFC scene	0.31 (0.30)	0.25 (0.15)	*−0.34* *(0.15)*	−0.17 (0.38)
EVC face	−0.37 (0.40)	0.32 (0.22)	*−0.58* *(0.21)*	0.21 (0.52)
EVC body^b^	−0.61 (0.55)	0.51 (0.38)	0.04 (0.37)	0.01 (0.75)
EVC object	**−1.89 (0.35)**	−0.02 (0.22)	−0.28 (0.21)	**1.48 (0.47)**
EVC scene^b^	*−0.62* *(0.33)*	0.12 (0.22)	*−0.44* *(0.22)*	0.32 (0.45)

a Parameters estimated with a linear mixed effect model in R. Condition responses indicated in the left column are the predictors, *z*-scored age coded as a fixed effect, and subject coded as a random effect. Standard error is indicated in parentheses. *p* < 0.05 is indicated in bold; *p* < 0.10 is indicated in italics.

b Model was singular due to negligible contribution of participant in the random effect term. A linear model without subject as a random effect produces the same results without a singular fit. Statistical models that included weights to account for the different amounts of data collected from each participant and each condition are reported in Table 3-1.

10.1523/ENEURO.0117-24.2024.t3-1 Table 3-1 **Effect of age on each condition with condition weights.** **^†^** Parameters estimated with a linear-mixed effects model in R. Condition response indicated in the left column are the predictors, z-scored age coded as a fixed effect, subject coded as a random effect. Standard error is indicated in paratheses. *p *< 0.05 is indicated in bold, *p *< 0.10 is indicated in italics. * Model was singular due to negligible contribution of participant in the random effects term. A linear model without subject as a random effect produces the same results without a singular fit. Statistical models without weights are reported in Table 3. Download Table 3-1, DOC file.

**Table 4.** Effect of age and hemisphere on face selectivity in each fROI

Variable	Sum Sq.	Num. DF	Den. DF	*F*	*p*
IOG					
Hemisphere	3.39	1	244.16	1.44	0.23
Condition	**105.06**	**3**	**244.16**	**14.94**	**0.000000006**
*Z*-Scored age	5.14	1	75.95	2.19	0.14
*Z*-Scored motion	*6*.*86*	*1*	*74*.*94*	*2.* *92*	*0.* *09*
Coil	1.59	1	37.97	0.68	0.42
Hemisphere * condition	1.83	3	244.16	0.26	0.85
Hemisphere * age	**10.21**	**1**	**244.16**	**4.36**	**0.04**
Condition * age	**21.64**	**3**	**244.16**	**3.08**	**0.03**
Hemi * condition * age	7.58	3	244.16	1.08	0.36
VTC					
Hemisphere	**28.98**	**1**	**248.87**	**11.77**	**0.0007**
Condition	**78.71**	**3**	**248.87**	**10.66**	**0.000001**
*Z*-Scored age	**21.33**	**1**	**52.54**	**8.67**	**0.0048**
*Z*-Scored motion	0.07	1	51.15	0.03	0.87
Coil	1.31	1	37.16	0.53	0.47
Hemisphere * condition	2.93	3	248.87	0.40	0.76
Hemisphere * age	0.58	1	248.87	0.24	0.63
Condition * age	12.10	3	248.87	1.64	0.18
Hemi * condition * age	4.13	3	248.87	0.56	0.64
STS					
Hemisphere	1.71	1	246.65	0.47	0.49
Condition	**86.44**	**3**	**246.65**	**7.94**	**0.00004**
*Z*-Scored age	1.87	1	99.03	0.52	0.47
*Z*-Scored motion	0.74	1	97.15	0.20	0.65
Coil	4.67	1	44.58	1.29	0.26
Hemisphere * condition	4.33	3	246.65	0.40	0.75
Hemisphere * age	*11.15*	*1*	*246.65*	*3.07*	*0.08*
Condition * age	**49.50**	**3**	**246.65**	**4.55**	**0.004**
Hemi * condition * age	2.73	3	246.65	0.25	0.86

All results from linear mixed effect models converted to ANOVA with R function anova; *p* < 0.05 is indicated in bold; *p* < 0.10 is indicated in italics. Statistics for models to test for face selectivity in each hemisphere are reported in Extended Data Table 4-1 (without weights) and Extended Data Table 4-2 (with weights). ANOVA tables from models testing for age by condition interactions in each fROI for each hemisphere are reported in Extended Data Table 2-3 (without weights) and Extended Data Table 2-4 (with weights). ANOVA tables for effects of age and hemisphere on face selectivity with weights to account for variable amounts of data collected from each infant and each condition are reported in Extended Data Table 4-3. Effects of age on each condition in each hemisphere are reported in Extended Data Table 4-4. Tests for interactions between hemisphere and age on each condition are reported in Extended Data Table 4-5.

10.1523/ENEURO.0117-24.2024.t4-1 Table 4-1 **Face selectivity in each fROI for each hemisphere.** Parameter estimates from linear mixed effects models with beta values for each condition as predictors. Indicator-coded vectors used to test if body, object, and scene responses are each significantly less than the response to faces. Sex and z-scored age were coded as fixed effects and subject was coded as a random effect. Standard error is indicated in paratheses; *p *< 0.05 is indicated in bold; *p *< 0.10 is indicated in italics. A negative number in bold indicates a significantly lower response to that condition to faces. The intercept indicates the magnitude of the face response relative to baseline. Models with weights in Table 4-2. Download Table 4-1, DOC file.

10.1523/ENEURO.0117-24.2024.t4-2 Table 4-2 **Face selectivity in fROI for each hemisphere with condition weights.** Parameter estimates from linear mixed effects models with beta values for each condition as predictors. Indicator-coded vectors used to test if body, object, and scene responses are each significantly less than the response to faces. Sex and z-scored age were coded as fixed effects and subject was coded as a random effect. Standard error is indicated in paratheses; *p *< 0.05 is indicated in bold; *p *< 0.10 is indicated in italics. A negative number in bold indicates a significantly lower response to that condition to faces. The intercept indicates the magnitude of the face response relative to baseline. Models without weights in Table 4-1. Download Table 4-2, DOC file.

10.1523/ENEURO.0117-24.2024.t4-3 Table 4-3 **Effect of age and hemisphere on face Selectivity in each fROI with condition weights.** All results from linear mixed effects models converted to ANOVA with R function anova; *p *< 0.05 is indicated in bold, *p *< 0.10 is indicated in italics. Statistics for models without weights are reported in Table 4. Download Table 4-3, DOC file.

10.1523/ENEURO.0117-24.2024.t4-4 Table 4-4 **Effect of Age on each condition in each hemisphere.** **^†^** Parameters estimated with a linear-mixed effects model in R. Condition response indicated in the left column are the predictors, z-scored age coded as a fixed effect, subject coded as a random effect. Standard error is indicated in paratheses. *p *< 0.05 is indicated in bold, *p *< 0.10 is indicated in italics. * Model was singular due to negligible contribution of participant in the random effects term. A linear model without subject as a random effect produces the same results without a singular fit. No weights included in analyses. Download Table 4-4, DOC file.

10.1523/ENEURO.0117-24.2024.t4-5 Table 4-5 **Effect of age and hemisphere on condition responses for each fROI.** All results from linear mixed effects models converted to ANOVA with R function anova; *p *< 0.05 is indicated in bold, *p *< 0.10 is indicated in italics. No weights included in analyses. Download Table 4-5, DOC file.

VTC is the location of FFA in adults. Across all infants, in the fROI in VTC (Fig. 5*b*; Table 1), the response to faces was significantly greater than responses to objects, bodies, and scenes (all *p*s < 0.007). The overall magnitude of response across all conditions increased with age (*p* = 0.01), but the age by condition interaction did not reach significance (*p* = 0.09; Table 2). Post hoc analyses of each condition separately (Extended Data Fig. 5-1; Table 3) showed that face and object responses were significantly greater in older infants (faces *p* = 0.04; objects *p* = 0.03; Extended Data Fig. 5-1; Table 3). In both the younger and older infants separately (Fig. 5*b*; Table 1), the face response was significantly greater than the response to bodies (younger, *p* = 0.001; older, *p* = 0.0009), objects (younger, *p* = 0.02; older, *p* = 0.04), and scenes (younger, *p* = 0.03; older, *p* = 0.00002). The response to all stimuli was higher overall in the right hemisphere (*p* = 0.0007; Table 4) and in older infants (*p* = 0.005), but there was no interaction between hemisphere and condition (*p* = 0.76) or hemisphere and age (*p* = 0.18). Post hoc analyses indicated there was no difference in responses to faces in right versus left hemispheres (*p* > 0.4; Fig. 6*a*; Tables 4–8).

Are the age effects observed in IOG stronger than the lack of age effects in VTC? To address this question, we tested for an fROI (IOG vs VTC) by age by condition interaction using a linear mixed effect model (Table 2). Indeed, although we observed a significant fROI (IOG vs VTC) by age interaction (*p* = 0.03; Table 2) and an interaction between condition and age (*p* = 0.0004), the fROI by age by condition interaction was not significant (*p* = 0.46).

STS contains a region that responds to both faces and voices in adults. Across all infants, in the fROI analysis of STS, the response to faces was significantly greater than to objects, bodies, and scenes (all *p*s < 0.00002; Fig. 5*c*; Table 1). There was no main effect of age (*p* = 0.7) and no condition by age interaction (*p* = 0.15; Table 2). In fROI analyses of the younger and older infants separately, the face response was significantly greater than responses to any other condition (all *p*s < 0.004). Overall, response magnitudes were higher in older infants (age by condition interaction, *p* = 0.004), but interactions with the hemisphere were not significant (hemisphere by age, *p* = 0.08; hemisphere by age by condition, *p* > 0.8).

In adults, MPFC contains a region that responds to socially relevant stimuli, including faces. Across all infants, in the fROI analysis of MPFC, the response to faces was significantly greater than to objects, bodies, and scenes (all *p*s < 0.00005; Fig. 5*d*; Table 1). There was no main effect of age (*p* = 0.7) and no age by condition interaction (*p* = 0.3; Table 2). In the younger infants alone, the MPFC face response was greater than the response to each other condition (bodies, *p* = 0.02; objects, *p* = 0.08; scenes, *p* = 0.005; all *p*s < 0.0002 in weighted LME; Table 1 and Extended Data Table 1-1). Older infants also had face-selective responses in MPFC (all *p*s < 0.00003), and responses to each condition did not change with age (all *p*s > 0.14; Extended Data Fig. 5-1; Table 3).

To test whether the face-selective responses described above are spatially specific to cortical regions with face-selective responses in adults, we conducted an fROI analysis in occipital areas, the location of early visual cortex (EVC) in adults, where we do not expect to observe face-selective responses. In the fROI analysis of EVC (Fig. 5*e*), the face response was significantly greater than the response to objects (*p* = 0.006), but it was numerically lower than the baseline (*p* = 0.06) and was not statistically different from body and scene responses (all *p*s > 0.1; Table 1). Further the face selectivity in IOG, VTC, and MPFC fROIs was significantly different from the absence of face selectivity in EVC (all *p*s < 0.05; Table 5). The condition by fROI responses for STS versus EVC did not reach significance (*p* = 0.30; Table 5). Thus, even in voxels selected for maximally face-selective responses, we confirmed that voxels in infant EVC are not face-selective and are significantly different from responses in IOG, VTC, and MPFC.

## Discussion

Here we test when face-selective cortical responses first arise in the approximate location of adult OFA, FFA, STS, and MPFC. We combined data collected with two different coils to double the sample size, compared with prior reports (Kosakowski et al., 2022a), and test whether face selectivity changes during the first year of life. To accommodate the different spatial distortions in the two coils, we used larger anatomical parcels, sacrificing spatial resolution in exchange for a larger sample of younger infants. Throughout the first year of life, including at the earliest ages we could measure, we observed face-selective responses in the approximate location of FFA, STS, and MPFC. In the approximate location of OFA, we did not observe face-selective responses in the youngest infants, but we did observe face-selective responses in older infants. Taken together, our results suggest that in humans, face-selective responses in multiple cortical regions emerge in infancy.

### OFA

The region in IOG, near adult OFA, appeared to show the most developmental change in our dataset. The region was face-selective on average in infants, and there was a significant age by condition interaction. In the older half of infants, we found face-selective responses, but not in the younger half of infants. This difference was driven by a decreased response to scenes in older infants, not a change in the response to faces.

The possibility that face selectivity might arise in OFA relatively later than in FFA is intriguing, particularly because OFA was initially presumed to be the source of face-specific input for FFA (Haxby et al., 2000; Gobbini and Haxby, 2007). Yet face selectivity is still observed in FFA following focal damage to OFA (Weiner et al., 2016; Gao et al., 2019; Rossion, 2022). Thus, it is debated whether OFA is a necessary source of input to FFA in a single hierarchy or whether FFA receives sufficient input from other sources (Pitcher et al., 2014; Pitcher, 2022; Rossion, 2022). The current results, finding face-selective responses in the youngest infants in FFA but not yet in OFA, could be construed as evidence supporting this latter idea.

However, the weaker response in the location of OFA in younger infants should be interpreted with caution. Even in adults, OFA is small, variable, and difficult to detect (Rossion et al., 2012; Zhen et al., 2015; Schwarz et al., 2019; Dai and Scherf, 2023). Although we could not confirm a face-selective response near OFA in the youngest infants, we also did not find strong evidence for its absence. More data from young infants will likely be needed to establish more precisely when a face-selective response can first be detected in IOG.

### FFA

Using the higher-resolution subset of these data collected with Coil 2021, a previous study reported that infants have face-selective responses in the approximate location of adult FFA (Kosakowski et al., 2022a). However, the sample was too small to test for developmental change within the first year. In the current analyses of the larger sample, we find no evidence that face selectivity is late to develop. Although older infants have a greater response to faces than younger infants, the youngest infants still have a detectable face-selective response in the approximate location of FFA, and there was no age by condition interaction.

The current results contribute to a long-standing debate about the origins of face selectivity in FFA. In children, FFA is face-selective, responding more to faces than to nonface visual categories (Passarotti et al., 2003; Aylward et al., 2005; Golarai et al., 2007, 2010, 2015; Scherf et al., 2007; Peelen et al., 2009; Pelphrey et al., 2009; Cantlon et al., 2011; Joseph et al., 2011; Natu et al., 2016; Dehaene-Lambertz et al., 2018; Nordt et al., 2021; Tian et al., 2021; Feng et al., 2022). However, compared with adults, children have (1) a smaller volume of the cortex with a significant face-selective response and (2) a smaller magnitude response difference between face and nonface categories (Golarai et al., 2007, 2010, 2015; Scherf et al., 2007; Peelen et al., 2009; Joseph et al., 2011; Haist et al., 2013; Natu et al., 2016; Nordt et al., 2021; Tian et al., 2021; Feng et al., 2022; although see Passarotti et al., 2003; Aylward et al., 2005; Dehaene-Lambertz et al., 2018). In children, the extent of face-selective cortex is correlated with face recognition and memory abilities (Golarai et al., 2007), which continue to develop into adulthood (Carey et al., 1992; Germine et al., 2011; Dundas et al., 2013).

Clearly, face-selective responses increase in extent and magnitude over childhood, but those observations do not establish when face-selective responses first emerge. The first neuroimaging studies with human infants suggested an early preferential response to faces in the approximate location of FFA, without full face selectivity. An early PET study demonstrated that young infants have responses to faces in the approximate location of FFA, STS, and MPFC (Tzourio-Mazoyer et al., 2002). But this study was limited because infants saw only two kinds of stimuli: static images of female faces and one control condition, colorful diodes. Similarly, an initial fMRI study found face preferences, but not selectivity, in a small sample of human infants (Deen et al., 2017). Consistent with evidence in humans, face-selective responses were observed in infant macaques only late in the first year of life (Livingstone et al., 2017), which is thought to correspond to approximately age 3 years in human development, and macaques that have never seen a face do not have face responses that are detectable with fMRI (Arcaro et al., 2017). Thus, initial PET and fMRI investigations in infants and children suggested that face selectivity in FFA might initially arise in toddlers or young children, only after substantial visual experience.

However, other fMRI and EEG data challenge that view. Two recent fMRI studies in awake infants observed face-selective responses in FFA, using different stimuli and task procedures (Kosakowski et al., 2022a; Yates et al., 2023). These results are consistent with EEG evidence of a distinctive response to faces, compared with that to other visual objects, in the brains of 4- to 6-month-old infants, with a source likely in VTC (De Heering and Rossion, 2015). Similarly, fMRI studies have found early origins for retinotopic organization of the visual cortex (Kourtzi et al., 2006; Ellis et al., 2021b) and for responses to motion in MT (Biagi et al., 2015, 2023). Compared with these other visual regions, FFA development might be less dependent on visual experience. Adults born with cataracts that were removed by age 2 have reduced motion-related responses in MT but have preserved responses in FFA (Guerreiro et al., 2022). Further, FFA responses are heritable (Polk et al., 2007; Chen et al., 2023) and are partially preserved in congenitally blind adults (Van Den Hurk et al., 2017; Ratan Murty et al., 2020). In sum, the current results fit with a growing body of evidence that face selectivity in FFA initially arises within a few months after birth and requires little visual experience.

Still, it is likely that many aspects of the FFA response change with both age and visual experience. The initial response to faces increases with age, even in infancy, and appears to expand to cover more of the fusiform gyrus during childhood (Golarai et al., 2007, 2010, 2015; Scherf et al., 2007; Peelen et al., 2009; Joseph et al., 2011; Natu et al., 2016; Nordt et al., 2021; Tian et al., 2021; Feng et al., 2022). Because of the spatial distortions, we cannot confidently estimate the size of the region we observed in infants. One important question for future research will be whether the size or selectivity of FFA in infants corresponds to their face recognition abilities.

### STS

A region in STS showed a robust response to faces compared with all other visual categories, in both the younger and older infants. The face stimuli were videos of children's faces, including changing facial expressions and gaze. In adults, these videos are ideal to elicit responses in STS, which strongly prefers dynamic to static faces (Sato et al., 2004; Pitcher et al., 2011, 2014, 2019). These results converge with prior evidence of face-selective responses in the approximate location of STS in children (Pitcher et al., 2011; Walbrin et al., 2020) and, using functional near–infrared spectroscopy (fNIRS), in infants (Lloyd-Fox et al., 2009; Farroni et al., 2013; Powell et al., 2018).

Although the STS responds selectively to faces among visual categories, in adults and older children, the same region also responds to other kinds of social stimuli. For example, the face-selective region in STS responds more to point-light displays depicting two bodies interacting versus two bodies not interacting (Isik et al., 2017; Walbrin et al., 2020). The same region responds more to human speech than nonspeech sounds (Deen et al., 2015). In infants, parts of STS similarly responds more to speech compared with nonspeech sounds (Grossmann et al., 2010; Lloyd-Fox et al., 2014) and point-light displays depicting biological versus non-biological motion (Lloyd-Fox et al., 2011; Lisboa et al., 2020a,b). However, it is not known whether these responses are colocated in the same regions of STS as the face-selective response.

The infants studied here, as young as 2 months, are among the youngest in whom STS responses to dynamic faces have been reported. A limitation of the current design is that we cannot test whether this same region already also responds to human voices or other social stimuli. It is an interesting developmental question whether responses to faces and voices are processed separately in early development and then gradually associated or whether these responses are already integrated within the first few months of life. Behaviorally, infants do seem to integrate face and voice information remarkably early. Young infants prefer to look at one face more than another based on nonvisual properties (e.g., speaker language, prosody, social behavior; Kelly et al., 2005; Kinzler et al., 2007; Turati et al., 2011). Even within the first 12 h after birth, infants prefer to look at their own mother's face, compared with a female stranger (Pascalis et al., 1995; Bushnell, 2001; Sai, 2005), identified by association with the mother's voice (Sai, 2005). In one series of studies, 3-h-old infants looked more at their mother's face than a female stranger's face, when they had experienced their mother's voice and face together in those 3 h, but not if their mother had been instructed to remain silent during those hours (Bushnell et al., 1989; Sai, 2005). Future research could investigate whether early developing multimodal responses in STS are related to infants’ behavioral preferences for faces associated with specific voices.

### Laterality of face responses

The robust lateralization of face responses to the right hemisphere in adults (Pitcher et al., 2007; Yovel et al., 2008; Rangarajan et al., 2014; Jonas et al., 2018) was not evident in our infant data, as none of the four face-selective regions showed a significant difference between hemispheres in the profile of response across conditions. Although this result could indicate that lateralization arises later in development (Behrmann and Plaut, 2020; Rossion and Lochy, 2022; Kubota et al., 2024), it is also possible that we simply lacked the power to detect effects of hemisphere (e.g., compare the nonsignificant trend in infant VTC in Fig. 6*b*). In the future, it will be important to use high-quality data from well-powered studies to determine when in development the lateralization of face selectivity in the right hemisphere emerges.

### MPFC

A region in MPFC showed face-selective responses in both younger and older infants that did not change with age in this sample. Finding selective functional responses in MPFC in infants as young as 2- to 5-months-old is intriguing in light of the protracted anatomical development of this region (Brody et al., 1987; Kinney et al., 1988; Tau and Peterson, 2010; Dubois et al., 2016; Vasung et al., 2019; Bethlehem et al., 2022). Signatures of cortical maturation including expansion (Li et al., 2013), increased sulcal depth (Meng et al., 2014), and myelination (Hasegawa et al., 1992; Carmody et al., 2004; Miller et al., 2012) occur relatively later in MPFC than in other cortical regions. Indeed, new neurons are still migrating and being integrated into the prefrontal cortex well into the second year of life (Sanai et al., 2011)—while this process is completed in primary sensory areas around the time of birth (Kostović et al., 2019).

In adults, a region in MPFC has been reported that responds more to images of faces than to other categories (Schwarz et al., 2019; Gu et al., 2023) and dynamic faces compared with dynamic objects similar to the current videos (Julian et al., 2012; Kosakowski et al., 2022b). Yet, the MPFC is not classically considered a face perception region (Haxby et al., 2000; Gobbini and Haxby, 2007) or a visual region, and its response to faces is modulated by the social content (LaBar, 2003; O’Doherty et al., 2003; Cheng et al., 2022). In addition, while OFA and FFA respond only to visually presented faces, face-selective regions in MPFC also respond to a variety of other social stimuli, including animations of social interactions and stories about people presented visually and aurally (Kosakowski et al., 2022b).

Similar to adults, studies using fNIRS have reported responses to dynamic faces in infant MPFC (Tzourio-Mazoyer et al., 2002; Grossmann et al., 2008; Krol and Grossmann, 2020; Porto et al., 2020; Farris et al., 2022). These responses are greater for socially relevant faces (e.g., a parent, direct gaze, using infant-directed speech) than faces with less social relevance (e.g., a diagram image, an averted gaze; Grossmann et al., 2008; Naoi et al., 2012; Imafuku et al., 2014; Lloyd-Fox et al., 2015; Urakawa et al., 2015; Xu et al., 2017; Uchida-Ota et al., 2019; Krol and Grossmann, 2020). One intriguing possibility is that infants’ MPFC, like adult MPFC, is engaged in processing the social and emotional meaning associated with faces. However, the current evidence cannot exclude the possibility that initially infants’ MPFC contains purely visual representations of faces and only later in infancy is used to ascribe social and emotional meaning to those faces.

Our results are broadly consistent with other recent evidence of functional responses in infants’ prefrontal cortex. For example, a region in the lateral prefrontal cortex in infants responds more to sequences with statistical regularity compared with the unstructured input (Gervain et al., 2008; Werchan et al., 2016; Ellis et al., 2021a). Another area in the infant prefrontal cortex responds more to native language compared with foreign language or other nonspeech sounds (Dehaene-Lambertz et al., 2010; Minagawa-kawai et al., 2011; Vouloumanos et al., 2010; May et al., 2011; Altvater-Mackensen and Grossmann, 2018). Thus, despite structural immaturity, the prefrontal cortex appears to be functionally active in infancy and may play a key role in infant cognitive development.

### Limitations and future directions

The current results provide an upper bound, but do not directly answer the question of when face selectivity first arises in each of the regions considered. Particularly in FFA, STS, and MPFC, face-selective responses are already present in the youngest group of infants, aged 2–5 months. We cannot resolve the time of first face-selective responses more specifically than this 3 month window, because we have limited data (average 16 min) from each infant. As a result, we cannot confidently estimate the selectivity of a region in a single infant. Moreover, we have no measurements in the first 2 months of infants’ lives, and so we cannot determine how much earlier face-selective responses first arise. These limitations leave open the intriguing possibility that face selectivity may not arise simultaneously across these cortical regions but, instead, arise in a sequence. A strong test of this hypothesis would ideally require substantially more data per infant, collected in a dense longitudinal sample, so that the age of first face-selective responses in each region could be confidently identified.

In order to increase the number of younger infants included in this sample, we combined data across two coils. The lower-resolution and more distorted images collected with Coil 2011 meant that we had to use very large parcels to identify voxels putatively near OFA and FFA in particular. Also, we did not have high-resolution anatomical images for most infants. As a result, the location of the fROIs reported here is approximate. Techniques for acquiring functional data from awake infants are improving rapidly (Cusack et al., 2018; Ellis et al., 2020; Yates et al., 2021), so the current results can be replicated in the future with greater confidence in the spatial origins of the measured signals.

Finally, although each of the regions tested showed a significantly greater response to faces than the other visual categories, the actual magnitude of the face responses was very small compared with those previously measured in children and adults. There are many possible explanations of the change in hemodynamic response magnitude over development, including both changes in neural firing rates and synchrony (Uhlhaas et al., 2010; Kiorpes, 2015) and changes in vasculature and neurovascular coupling (Colonnese et al., 2008). The current study cannot differentiate between these explanations. However, the change in magnitude we observed between age 2 and 9 months was gradual and moderate, consistent with other evidences that the magnitude of hemodynamic responses change slowly and gradually throughout infancy and childhood (Cohen Kadosh et al., 2011, 2013; Arichi et al., 2012; Cusack et al., 2015; De Oliveira et al., 2017).

### Summary

In sum, using fMRI data from a large sample of awake infants, we measured face-selective responses in multiple regions of infants’ brains. Robust face selectivity was present in the approximate location of FFA, STS, and MPFC as early as we could measure. Putative OFA also had face-selective responses in older infants but not yet younger infants. Despite undergoing rapid anatomical change in the first postnatal year, the infant cortex already has structured responses to meaningful, self-relevant stimuli such as faces. These results importantly constrain theories of cortical development and the origins of face selectivity.

## Synthesis

Reviewing Editor: Ifat Levy, Yale School of Medicine Decisions are customarily a result of the Reviewing Editor and the peer reviewers coming together and discussing their recommendations until a consensus is reached. When revisions are invited, a fact-based synthesis statement explaining their decision and outlining what is needed to prepare a revision will be listed below. The following reviewer(s) agreed to reveal their identity: NONE.

This manuscript reports rare awake infant fMRI data from a large sample of infants, contributing to a small but growing field that aims to understand the neurodevelopmental origins of cognition. This study represents a significant and ambitious effort to explore the developmental onset of face selectivity in various cortical regions of the infant brain, a topic of substantial importance in understanding the early mechanisms of visual recognition and social cognition. The successful collection of functional magnetic resonance imaging (fMRI) data from such a large sample of infants, aged 2-9 months, is commendable, given the inherent challenges in scanning this population. The findings of significant selectivity across the brain even in the youngest infants tested, potentially contribute valuable insights into the constraints on theories of cortical development. The literature reviewed in the Introduction and Discussion is thorough and well-written, and the results are appropriately discussed with respect to limitations and the need for future work.

Reviewers had, however, several comments which need to be addressed, as described below:

- The authors need to more clearly delineate which analyses are novel and which are extensions of the prior work (Kosakowski et al., 2022). The claim of doubling the sample size compared to prior reports warrants a more transparent comparison and justification, particularly considering the inclusion of infants from two coils in the earlier study, which also reported on a substantial sample size. In particular, Kosakowski et al., reports on useable data from 52 infants (26 from each coil) compared to 65 infants for the current study. The previous paper already reported evidence of face selectivity in the FFA and OFA in these infants. There are some important differences in the previously reported analyses and the ones reported here - e.g., whether the data was combined by Coil, the size of the ROIs, and the breakdown by age - but also several similarities. For instance, Figure 4 in the current paper shows group effects for the Coil 2021 data, which is seemingly also what is shown in Figure 2 in Kosakowski et al. 2022. If this assessment is correct, this should be clearly acknowledged in the figure caption. In general, it may help to frame the ROI results for FFA and OFA as a check of the robustness of the prior results, with similar findings in a larger ROI search space, while the STS and mPFC analyses are unique to the current paper.

- A related point is that the authors note several times in the manuscript that there was considerable distortion in the images acquired in the Coil 2011 dataset compared to the Coil 2021 dataset. This makes it curious as to why the authors combined the two datasets. The reasoning given is that it increases the sample size to allow for age-related analyses. However, it would be helpful if the authors could provide more justification for why they believe combining these two datasets is appropriate and valid. For example, some measure of comparable data quality (e.g., signal to noise levels) may help increase confidence in this approach.

- One analysis that could help with the concern raised in the previous point is the supplemental linear mixed effects model with Coil as a random effect, which did not change the overall pattern of their results. However, reviewers wondered if the appropriate way to model the effect of Coil is not as a random effect, but as a predictor instead, perhaps with interaction terms as well. This is important because the prior study using this same dataset found a significant coil by stimulus condition by fROI interaction (p. 267 of Kosakowski et al., 2022). At present, it is a little confusing as to why Coil mattered in the prior study but not in this one, especially since similar ROIs were used across these two papers.

- In the Abstract, it is noted that: "individual subject whole-brain activations maps revealed a significant response to faces compared to non-face visual categories in the approximate location of OFA, FFA, STS, and MPFC." Two such maps are visualized in Figure 3. However, the number of individual subjects' maps in which these regions could be found is not reported (in the main text on p. 11 it just says "many infants... from both the Coil 2021 and Coil 2011 datasets"). If the authors want to make this kind of claim regarding the whole brain analyses, it would be important to provide some quantitative data - i.e., the authors could count the number of infants with significant activation to faces > non-faces in the approximate locations of the OFA, FFA, etc. (perhaps using the ROIs from the fROI analysis as guides). Given that there were weaker whole brain results for the Coil 2011 group in the supplement, it would be interesting to know if most Coil 2011 infants still showed face > non-face responses in their individual maps (perhaps giving more confidence that weaker effects in the whole brain are due to variability in localization/registration across subjects, rather than a possible lack of face selectivity).

- The inclusion of the EVC as a control region is aimed to demonstrate that it is not guaranteed to get significant face selectivity using the fROI approach. However, these results are not plotted in the main text or the supplement. In addition, this area also shows some preference to faces, as it responds more strongly to faces vs. objects. To show that the findings indeed reflect face selectivity in specific areas, rather than a broader visual cortex bias towards faces, it's crucial to include a control region in high-level visual cortex (e.g., a place-selective region).

- The finding that face selectivity is bilateral early in life is really interesting in the context of debates surrounding how category selective regions emerge over development and why we see laterality in adults (e.g., Berhmann &Plaut, 2020, Perception; Kubota et al., 2023, TICS). The authors should consider including some reference to this literature in the Discussion, even if the current results do not resolve this debate.

- In the discussion the authors point to a potential difference in the maturation rate of the FFA and the OFA. However, A more detailed analysis examining the interaction between age, condition, and region, is necessary to substantiate claims about their distinct developmental paths.

- While it is clear that the sub-runs approach is needed to generate useable data from infants, this approach also has some limitations that should be acknowledged. For example, sub-runs that are created from "gluing" together mini sub-runs are likely to be noisier and variable compared with a coherent sub-run. The authors should include an analysis that demonstrates that this is not case (or if this is the case, address it statistically)

Minor comments

- Figures 4 and 4-1: The Methods section states that 49 sessions went into the whole-brain analyses, but the analyses were conducted for Coil 2021 and Coil 2011 separately. How many infants went into each of these group analyses? The authors should add this information to the figure captions.

- p. 17: the Li et al. 2022 citation is referenced in a misleading way. This paper found that cortical myelination in ventral visual cortex seems to be more related to gestational age (i.e., endogenous/innate scaffolding), but cortical thickness and functional connectivity were more related to postnatal time, suggesting the importance of visual experience. This latter finding with respect to function is probably more relevant to the current study than myelination.

- The discussion insufficiently addresses the significant body of literature indicating the necessity of visual experience for the emergence of face selectivity, including works by Arcaro et al., 2017, and related studies. Acknowledgment of these studies would enrich the discourse on the development of face-selective regions.

- Note that the original images in Figure 1a + b are absent - this may be intentional at this stage, but should be fixed before publication.

## Author Response

Thanks to both you and the reviewers for helpful comments on our manuscript, "Cortical Face-Selective Responses Emerge Early in Human Infancy" (MS No. eN- NWR-0117-24R1). Please find attached a revision of our manuscript. We have incorporated many of these suggestions made by the reviewers and carried out several new analyses to address their suggestions and concerns. Overall, we believe these changes improved the manuscript and we hope that you agree that the paper is now suitable for publication in eNeuro. Below, we go point-by-point in describing our responses/changes to every issue raised by the reviewers.

Synthesis of Reviews:

Synthesis Statement for Author (Required):

This manuscript reports rare awake infant fMRI data from a large sample of infants, contributing to a small but growing field that aims to understand the neurodevelopmental origins of cognition. This study represents a significant and ambitious effort to explore the developmental onset of face selectivity in various cortical regions of the infant brain, a topic of substantial importance in understanding the early mechanisms of visual recognition and social cognition. The successful collection of functional magnetic resonance imaging (fMRI) data from such a large sample of infants, aged 2-9 months, is commendable, given the inherent challenges in scanning this population. The findings of significant selectivity across the brain even in the youngest infants tested, potentially contribute valuable insights into the constraints on theories of cortical development. The literature reviewed in the Introduction and Discussion is thorough and well-written, and the results are appropriately discussed with respect to limitations and the need for future work.

Reviewers had, however, several comments which need to be addressed, as described below:

- The authors need to more clearly delineate which analyses are novel and which are extensions of the prior work (Kosakowski et al., 2022). The claim of doubling the sample size compared to prior reports warrants a more transparent comparison and justification, particularly considering the inclusion of infants from two coils in the earlier study, which also reported on a substantial sample size. In particular, Kosakowski et al., reports on useable data from 52 infants (26 from each coil) compared to 65 infants for the current study. The previous paper already reported evidence of face selectivity in the FFA and OFA in these infants. There are some important differences in the previously reported analyses and the ones reported here - e.g., whether the data was combined by Coil, the size of the ROIs, and the breakdown by age - but also several similarities. For instance, Figure 4 in the current paper shows group effects for the Coil 2021 data, which is seemingly also what is shown in Figure 2 in Kosakowski et al. 2022. If this assessment is correct, this should be clearly acknowledged in the figure caption. In general, it may help to frame the ROI results for FFA and OFA as a check of the robustness of the prior results, with similar findings in a larger ROI search space, while the STS and mPFC analyses are unique to the current paper.

Thank you for bringing up this matter. We have added a table to the supplementary materials to with footnotes to explain deviations from Kosakowski et al., 2022.

- A related point is that the authors note several times in the manuscript that there was considerable distortion in the images acquired in the Coil 2011 dataset compared to the Coil 2021 dataset. This makes it curious as to why the authors combined the two datasets. The reasoning given is that it increases the sample size to allow for age-related analyses. However, it would be helpful if the authors could provide more justification for why they believe combining these two datasets is appropriate and valid. For example, some measure of comparable data quality (e.g., signal to noise levels) may help increase confidence in this approach.

The main disadvantage of combining data across coils is that the necessary larger parcels makes it harder to be confident exactly where, spatially, the observed responses arise in cortex. Thus, compared to the previous paper which relied on Coil2021, we can be less confident that the responses we measured in 'VTC' definitely arise from the location of adult FFA. The previous paper was focused on the comparison between adult regions of interest and activity in infants, for which better spatial resolution is important. The current analyses are focused comparisons between infants, across age and cortical lobe. For these analyses, spatial precision was less important than having a large enough sample size to test for selectivity in the younger half of infants, aged 2-5 months, which we could only do by combining data across coils.

- One analysis that could help with the concern raised in the previous point is the supplemental linear mixed effects model with Coil as a random effect, which did not change the overall pattern of their results. However, reviewers wondered if the appropriate way to model the effect of Coil is not as a random effect, but as a predictor instead, perhaps with interaction terms as well. This is important because the prior study using this same dataset found a significant coil by stimulus condition by fROI interaction (p. 267 of Kosakowski et al., 2022). At present, it is a little confusing as to why Coil mattered in the prior study but not in this one, especially since similar ROIs were used across these two papers.

This point is well taken and we will use models with coil as a fixed predictor.

- In the Abstract, it is noted that: "individual subject whole-brain activations maps revealed a significant response to faces compared to non-face visual categories in the approximate location of OFA, FFA, STS, and MPFC." Two such maps are visualized in Figure 3. However, the number of individual subjects' maps in which these regions could be found is not reported (in the main text on p. 11 it just says "many infants... from both the Coil 2021 and Coil 2011 datasets"). If the authors want to make this kind of claim regarding the whole brain analyses, it would be important to provide some quantitative data - i.e., the authors could count the number of infants with significant activation to faces > non-faces in the approximate locations of the OFA, FFA, etc. (perhaps using the ROIs from the fROI analysis as guides). Given that there were weaker whole brain results for the Coil 2011 group in the supplement, it would be interesting to know if most Coil 2011 infants still showed face > non-face responses in their individual maps (perhaps giving more confidence that weaker effects in the whole brain are due to variability in localization/registration across subjects, rather than a possible lack of face selectivity).

We have added whole-brain activation maps for each datapoint from each infant. We used red circles to highlight the approximate location of each region, even if there are no observable activations in those regions. These images highlight the di\erential distortions, the variability in activity, but importantly show that many infants had activations in each region.

- The inclusion of the EVC as a control region is aimed to demonstrate that it is not guaranteed to get significant face selectivity using the fROI approach. However, these results are not plotted in the main text or the supplement. In addition, this area also shows some preference to faces, as it responds more strongly to faces vs. objects. To show that the findings indeed reflect face selectivity in specific areas, rather than a broader visual cortex bias towards faces, it's crucial to include a control region in high-level visual cortex (e.g., a place-selective region).

We have added a plot of the EVC results to the main text. As you can see, although the face response in EVC is signiﬁcantly greater than the response to objects, is not signiﬁcantly di\erent from the response to the baseline condition, bodies, or scenes. Thus, the face response in EVC is not face-selective. Further, there is a signiﬁcant interaction between EVC and IOG, VTC, &MPFC further demonstrating that the response in EVC is not face selective.

We would also refer the reviewers to Kosakowski et al., 2022 Fig 6 which demonstrates that place and body regions do not have face-selective responses.

- The finding that face selectivity is bilateral early in life is really interesting in the context of debates surrounding how category selective regions emerge over development and why we see laterality in adults (e.g., Berhmann &Plaut, 2020, Perception; Kubota et al., 2023, TICS). The authors should consider including some reference to this literature in the Discussion, even if the current results do not resolve this debate.

Thank you, we have added these citations to the discussion.

- In the discussion the authors point to a potential difference in the maturation rate of the FFA and the OFA. However, A more detailed analysis examining the interaction between age, condition, and region, is necessary to substantiate claims about their distinct developmental paths.

We have added an ANOVA to test for the three-way interaction. It is important to note that we raise it as a possibility that there are di\erential rates of development rates for FFA and OFA but also acknowledge the need for more data to substantiate such a claim. Speciﬁcally, in the discussion we state, "Although we could not conﬁrm a face- selective response near OFA in the youngest infants, we also did not ﬁnd strong evidence for its absence. More data from young infants will likely be needed to establish more precisely when a face-selective response can ﬁrst be detected in inferior occipital gyrus." - While it is clear that the sub-runs approach is needed to generate useable data from infants, this approach also has some limitations that should be acknowledged. For example, sub-runs that are created from "gluing" together mini sub-runs are likely to be noisier and variable compared with a coherent sub-run. The authors should include an analysis that demonstrates that this is not case (or if this is the case, address it statistically) The question of whether runs should be concatenated or averaged has been extensively addressed by the fMRI community. We used the guidelines set forth in Handbook of Functional FMRI Data Analysis by Poldrack, Mumford, and Nichols that noted concatenating runs increases statistical power over averaged runs. However, as the reviewer notes, these guidelines are made assuming (1) low-motion fMRI data and (2) a balanced design (i.e., the same number of volumes in each run). Here, although we did attempt to account for motion and attempt to balance the design in the fROI analyses, we did not statistically interrogate the impact of concatenating infant fMRI data. Thus, to address the reviewers' concerns, we computed the temporal signal to noise ratio (tSNR) in each data partition that we used as an analytic component and updated the methods section with the results. Thank you for this suggestion, we think it improves the quality of our work.

Minor comments - Figures 4 and 4-1: The Methods section states that 49 sessions went into the whole-brain analyses, but the analyses were conducted for Coil 2021 and Coil 2011 separately. How many infants went into each of these group analyses? The authors should add this information to the figure captions.

This information has been added to a table in SM.

- p. 17: the Li et al. 2022 citation is referenced in a misleading way. This paper found that cortical myelination in ventral visual cortex seems to be more related to gestational age (i.e., endogenous/innate scaffolding), but cortical thickness and functional connectivity were more related to postnatal time, suggesting the importance of visual experience. This latter finding with respect to function is probably more relevant to the current study than myelination.

To address the reviewers' concerns, we have removed this citation.

- The discussion insufficiently addresses the significant body of literature indicating the necessity of visual experience for the emergence of face selectivity, including works by Arcaro et al., 2017, and related studies. Acknowledgment of these studies would enrich the discourse on the development of face-selective regions.

We have added Arcaro et al., 2017.

- Note that the original images in Figure 1a + b are absent - this may be intentional at this stage, but should be fixed before publication.

## References

[B1] Altvater-Mackensen N, Grossmann T (2018) Modality-independent recruitment of inferior frontal cortex during speech processing in human infants. Dev Cogn Neurosci 34:130–138. 10.1016/j.dcn.2018.10.002 30391756 PMC6969291

[B2] Arcaro MJ, Schade PF, Vincent JL, Ponce CR, Livingstone MS (2017) Seeing faces is necessary for face-domain formation. Nat Neurosci 20:1404–1412. 10.1038/nn.4635 28869581 PMC5679243

[B3] Arichi T, et al. (2012) Development of BOLD signal hemodynamic responses in the human brain. Neuroimage 63:663–673. 10.1016/j.neuroimage.2012.06.054 22776460 PMC3459097

[B4] Axelrod V, Rozier C, Malkinson TS, Lehongre K, Adam C, Lambrecq V, Navarro V, Naccache L (2019) Face-selective neurons in the vicinity of the human fusiform face area. Neurology 92:197–198. 10.1212/WNL.0000000000006806 30665911

[B5] Aylward EH, Park JE, Field KM, Parsons AC, Richards TL, Cramer SC, Meltzoff AN (2005) Brain activation during face perception: evidence of a developmental change. J Cogn Neurosci 17:308–319. 10.1162/0898929053124884 15811242

[B6] Bates D, Mächler M, Bolker B, Walker S (2014) Fitting linear mixed-effects models using lme4. arXiv preprint arXiv:1406.5823.

[B7] Behrmann M, Plaut DC (2020) Hemispheric organization for visual object recognition: a theoretical account and empirical evidence. Perception 49:373–404. 10.1177/0301006619899049 31980013 PMC9944149

[B8] Bethlehem RAI, et al. (2022) Brain charts for the human lifespan. Nature 604:525–533. 10.1038/s41586-022-04554-y 35388223 PMC9021021

[B9] Biagi L, Crespi SA, Tosetti M, Morrone MC (2015) BOLD response selective to flow-motion in very young infants. PLoS Biol 13:e1002260. 10.1371/journal.pbio.1002260 26418729 PMC4587790

[B10] Biagi L, Tosetti M, Crespi SA, Morrone MC (2023) Development of BOLD response to motion in human infants. J Neurosci 43:3825–3837. 10.1523/JNEUROSCI.0837-22.2023 37037605 PMC10218011

[B11] Brody BA, Kinney HC, Kloman AS, Gilles FH (1987) Sequence of central nervous system myelination in human infancy. J Neuropathol Exp Neurol 46:283–301. 10.1097/00005072-198705000-00005 3559630

[B12] Bushnell IWR (2001) Mother’s face recognition in newborn infants: learning and memory. Infant Child Dev 10:67–74. 10.1002/icd.248

[B13] Bushnell IWR, Sai F, Mullin JT (1989) Neonatal recognition of the mother’s face. Br J Dev Psychol 7:3–15. 10.1111/j.2044-835X.1989.tb00784.x

[B14] Cantlon JF, Pinel P, Dehaene S, Pelphrey KA (2011) Cortical representations of symbols, objects, and faces are pruned back during early childhood. Cereb Cortex 21:191–199. 10.1093/cercor/bhq078 20457691 PMC3000569

[B15] Carey S, De Schonen S, Ellis HD (1992) Becoming a face expert. Philos Trans R Soc B Biol Sci 335:95–103. 10.1098/rstb.1992.0012 1348143

[B16] Carmody DP, Dunn SM, Boddie-Willis AS, DeMarco JK, Lewis M (2004) A quantitative measure of myelination development in infants, using MR images. Neuroradiology 46:781–786. 10.1007/s00234-004-1241-z 15243725 PMC1513122

[B17] Chen X, Liu X, Parker BJ, Zhen Z, Weiner KS (2023) Functionally and structurally distinct fusiform face area(s) in over 1000 participants. Neuroimage 265:119765. 10.1016/j.neuroimage.2022.119765 36427753 PMC9889174

[B18] Cheng Q, Han Z, Liu S, Kong Y, Weng X, Mo L (2022) Neural responses to facial attractiveness in the judgments of moral goodness and moral beauty. Brain Struct Funct 227:843–863. 10.1007/s00429-021-02422-5 34767078

[B19] Cohen Kadosh K, Cohen Kadosh R, Dick F, Johnson MH (2011) Developmental changes in effective connectivity in the emerging core face network. Cereb Cortex 21:1389–1394. 10.1093/cercor/bhq215 21045001 PMC3094719

[B20] Cohen Kadosh K, Johnson MH, Henson RNA, Dick F, Blakemore S-J (2013) Differential face-network adaptation in children, adolescents and adults. Neuroimage 69:11–20. 10.1016/j.neuroimage.2012.11.060 23231884

[B21] Colonnese MT, Phillips MA, Constantine-Paton M, Kaila K, Jasanoff A (2008) Development of hemodynamic responses and functional connectivity in rat somatosensory cortex. Nat Neurosci 11:72–79. 10.1038/nn2017 18037883

[B22] Cousineau D (2005) Confidence intervals in within-subject designs: a simpler solution to Loftus and Masson’s method. Tutor Quant Methods Psychol 1:42–45. 10.20982/tqmp.01.1.p042

[B23] Cusack R, McCuaig O, Linke AC (2018) Methodological challenges in the comparison of infant fMRI across age groups. Dev Cogn Neurosci 33:194–205. 10.1016/j.dcn.2017.11.003 29158073 PMC6969274

[B24] Cusack R, Wild C, Linke AC, Arichi T, Lee DSC, Han VK (2015) Optimizing stimulation and analysis protocols for neonatal fMRI. PLoS One 10:e0120202. 10.1371/journal.pone.0120202 26266954 PMC4534447

[B25] Dai J, Scherf KS (2023) The privileged status of peer faces: subordinate-level neural representations of faces in emerging adults. J Cogn Neurosci 35:715–735. 10.1162/jocn_a_01966 36638228 PMC12363370

[B26] Deen B, Koldewyn K, Kanwisher N, Saxe R (2015) Functional organization of social perception and cognition in the superior temporal sulcus. Cereb Cortex 25:4596–4609. 10.1093/cercor/bhv111 26048954 PMC4816802

[B27] Deen B, Richardson H, Dilks DD, Takahashi A, Keil B, Wald LL, Kanwisher N, Saxe R (2017) Organization of high-level visual cortex in human infants. Nat Commun 8:13995. 10.1038/ncomms13995 28072399 PMC5234071

[B28] Deen B, Saxe R, Kanwisher N (2020) Processing communicative facial and vocal cues in the superior temporal sulcus. Neuroimage 221:117191. 10.1016/j.neuroimage.2020.117191 32711066

[B29] Dehaene-Lambertz G, Montavont A, Jobert A, Allirol L, Dubois J, Hertz-Pannier L, Dehaene S (2010) Language or music, mother or Mozart? Structural and environmental influences on infants’ language networks. Brain Lang 114:53–65. 10.1016/j.bandl.2009.09.003 19864015

[B30] Dehaene-Lambertz G, Monzalvo K, Dehaene S (2018) The emergence of the visual word form: longitudinal evolution of category-specific ventral visual areas during reading acquisition. PLoS Biol 16:e2004103. 10.1371/journal.pbio.2004103 29509766 PMC5856411

[B31] De Heering A, Rossion B (2015) Rapid categorization of natural face images in the infant right hemisphere. Elife 4:e06564. 10.7554/eLife.06564 26032564 PMC4450157

[B32] De Oliveira SR, De Paula Machado ACC, De Paula JJ, De Moraes PHP, Nahin MJS, Magalhães LDC, Novi SL, Mesquita RC, De Miranda DM, Bouzada MCF (2017) Association between hemodynamic activity and motor performance in six-month-old full-term and preterm infants: a functional near-infrared spectroscopy study. Neurophotonics 5:011016. 10.1117/1.NPh.5.1.011016 29057284 PMC5637226

[B33] Dubois J, Poupon C, Thirion B, Simonnet H, Kulikova S, Leroy F, Hertz-Pannier L, Dehaene-Lambertz G (2016) Exploring the early organization and maturation of linguistic pathways in the human infant brain. Cereb Cortex 26:2283–2298. 10.1093/cercor/bhv082 25924951

[B34] Dundas EM, Plaut DC, Behrmann M (2013) The joint development of hemispheric lateralization for words and faces. J Exp Psychol Gen 142:348–358. 10.1037/a0029503 22866684 PMC4241688

[B35] Ellis CT, Skalaban LJ, Yates TS, Bejjanki VR, Córdova NI, Turk-Browne NB (2020) Re-imagining fMRI for awake behaving infants. Nat Commun 11:4523. 10.1038/s41467-020-18286-y 32908125 PMC7481790

[B36] Ellis CT, Skalaban LJ, Yates TS, Turk-Browne NB (2021a) Attention recruits frontal cortex in human infants. Proc Natl Acad Sci U S A 118:e2021474118. 10.1073/pnas.2021474118 33727420 PMC7999871

[B37] Ellis CT, Yates TS, Skalaban LJ, Bejjanki VR, Arcaro MJ, Turk-Browne NB (2021b) Retinotopic organization of visual cortex in human infants. Neuron 109:2616–2626.e6. 10.1016/j.neuron.2021.06.004 34228960

[B38] Farris K, Kelsey CM, Krol KM, Thiele M, Hepach R, Haun DB, Grossmann T (2022) Processing third-party social interactions in the human infant brain. Infant Behav Dev 68:101727. 10.1016/j.infbeh.2022.101727 35667276

[B39] Farroni T, Chiarelli AM, Lloyd-Fox S, Massaccesi S, Merla A, Di Gangi V, Mattarello T, Faraguna D, Johnson MH (2013) Infant cortex responds to other humans from shortly after birth. Sci Rep 3:2851. 10.1038/srep02851 24092239 PMC3790196

[B40] Feng X, Monzalvo K, Dehaene S, Dehaene-Lambertz G (2022) Evolution of reading and face circuits during the first three years of reading acquisition. Neuroimage 259:119394. 10.1016/j.neuroimage.2022.119394 35718022

[B41] Gao X, Vuong QC, Rossion B (2019) The cortical face network of the prosopagnosic patient PS with fast periodic stimulation in fMRI. Cortex 119:528–542. 10.1016/j.cortex.2018.11.008 30545601

[B42] Gauthier I, Tarr MJ, Moylan J, Skudlarski P, Gore JC, Anderson AW (2000) The fusiform “face area” is part of a network that processes faces at the individual level. J Cogn Neurosci 12:495–504. 10.1162/089892900562165 10931774

[B43] Germine LT, Duchaine B, Nakayama K (2011) Where cognitive development and aging meet: face learning ability peaks after age 30. Cognition 118:201–210. 10.1016/j.cognition.2010.11.002 21130422

[B44] Gervain J, Macagno F, Cogoi S, Peña M, Mehler J (2008) The neonate brain detects speech structure. Proc Natl Acad Sci U S A 105:14222–14227. 10.1073/pnas.0806530105 18768785 PMC2544605

[B45] Ghotra A, et al. (2021) A size-adaptive 32-channel array coil for awake infant neuroimaging at 3 Tesla MRI. Magn Reson Med 86:1773–1785. 10.1002/mrm.28791 33829546

[B46] Glasser MF, et al. (2016) A multi-modal parcellation of human cerebral cortex. Nature 536:171–178. 10.1038/nature18933 27437579 PMC4990127

[B47] Gobbini MI, Haxby JV (2007) Neural systems for recognition of familiar faces. Neuropsychologia 45:32–41. 10.1016/j.neuropsychologia.2006.04.015 16797608

[B48] Golarai G, Ghahremani DG, Whitfield-Gabrieli S, Reiss A, Eberhardt JL, Gabrieli JDE, Grill-Spector K (2007) Differential development of high-level visual cortex correlates with category-specific recognition memory. Nat Neurosci 10:512–522. 10.1038/nn1865 17351637 PMC3660101

[B49] Golarai G, Liberman A, Grill-Spector K (2015) Experience shapes the development of neural substrates of face processing in human ventral temporal cortex. Cereb Cortex 27:1229–1244. 10.1093/cercor/bhv314 26683171 PMC6161183

[B50] Golarai G, Liberman A, Yoon J, Grill-Spector K (2010) Differential development of the ventral visual cortex extends through adolescence. Front Hum Neurosci 3:1057. 10.3389/neuro.09.080.2009 20204140 PMC2831628

[B51] Grill-Spector K, Knouf N, Kanwisher N (2004) The fusiform face area subserves face perception, not generic within-category identification. Nat Neurosci 7:555–562. 10.1038/nn1224 15077112

[B52] Grossmann T, Johnson MH, Lloyd-Fox S, Blasi A, Deligianni F, Elwell C, Csibra G (2008) Early cortical specialization for face-to-face communication in human infants. Proc R Soc B Biol Sci 275:2803–2811. 10.1098/rspb.2008.0986 18755668 PMC2572680

[B53] Grossmann T, Oberecker R, Koch SP, Friederici AD (2010) The developmental origins of voice processing in the human brain. Neuron 65:852–858. 10.1016/j.neuron.2010.03.001 20346760 PMC2852650

[B54] Gu L, Li A, Yang R, Yang J, Pang Y, Qu J, Mei L (2023) Category-specific and category-general neural codes of recognition memory in the ventral visual pathway. Cortex 164:77–89. 10.1016/j.cortex.2023.04.004 37207411

[B55] Guerreiro MJS, Kekunnaya R, Röder B (2022) Top-down modulation of visual cortical processing after transient congenital blindness. Neuropsychologia 174:108338. 10.1016/j.neuropsychologia.2022.108338 35931134

[B56] Haist F, Adamo M, Han Wazny J, Lee K, Stiles J (2013) The functional architecture for face-processing expertise: FMRI evidence of the developmental trajectory of the core and the extended face systems. Neuropsychologia 51:2893–2908. 10.1016/j.neuropsychologia.2013.08.005 23948645 PMC3825803

[B57] Hasegawa M, Houdou S, Mito T, Takashima S, Asanuma K, Ohno T (1992) Development of myelination in the human fetal and infant cerebrum: a myelin basic protein immunohistochemical study. Brain Dev 14:1–6. 10.1016/S0387-7604(12)80271-3 1375444

[B58] Haxby JV, Hoffman EA, Gobbini MI (2000) The distributed human neural system for face perception. Trends Cogn Sci 4:223–233. 10.1016/S1364-6613(00)01482-0 10827445

[B59] Henriksson L, Mur M, Kriegeskorte N (2015) Faciotopy—a face-feature map with face-like topology in the human occipital face area. Cortex 72:156–167. 10.1016/j.cortex.2015.06.030 26235800 PMC4643680

[B60] Ichikawa H, Kanazawa S, Yamaguchi MK, Kakigi R (2010) Infant brain activity while viewing facial movement of point-light displays as measured by near-infrared spectroscopy (NIRS). Neurosci Lett 482:90–94. 10.1016/j.neulet.2010.06.086 20609380

[B61] Imafuku M, Hakuno Y, Uchida-Ota M, Yamamoto J, Minagawa Y (2014) “Mom called me!” Behavioral and prefrontal responses of infants to self-names spoken by their mothers. Neuroimage 103:476–484. 10.1016/j.neuroimage.2014.08.034 25175541

[B62] Isik L, Koldewyn K, Beeler D, Kanwisher N (2017) Perceiving social interactions in the posterior superior temporal sulcus. Proc Natl Acad Sci U S A 114:E9145–E9152. 10.1073/pnas.1714471114 29073111 PMC5664556

[B63] Jonas J, Brissart H, Hossu G, Colnat-Coulbois S, Vignal J-P, Rossion B, Maillard L (2018) A face identity hallucination (palinopsia) generated by intracerebral stimulation of the face-selective right lateral fusiform cortex. Cortex 99:296–310. 10.1016/j.cortex.2017.11.022 29306709

[B64] Joseph JE, Gathers AD, Bhatt RS (2011) Progressive and regressive developmental changes in neural substrates for face processing: testing specific predictions of the interactive specialization account: developmental changes in face processing. Dev Sci 14:227–241. 10.1111/j.1467-7687.2010.00963.x 21399706 PMC3050484

[B65] Julian JB, Fedorenko E, Webster J, Kanwisher N (2012) An algorithmic method for functionally defining regions of interest in the ventral visual pathway. Neuroimage 60:2357–2364. 10.1016/j.neuroimage.2012.02.055 22398396

[B66] Kanwisher N, McDermott J, Chun MM (1997) The fusiform face area: a module in human extrastriate cortex specialized for face perception. J Neurosci 7:4302–4311. 10.1523/JNEUROSCI.17-11-04302.1997 9151747 PMC6573547

[B67] Kay KN, Rokem A, Winawer J, Dougherty RF, Wandell BA (2013) GLMdenoise: a fast, automated technique for denoising task-based fMRI data. Front Neurosci 7:247. 10.3389/fnins.2013.00247 24381539 PMC3865440

[B68] Keil B, et al. (2011) Size-optimized 32-channel brain arrays for 3T pediatric imaging: pediatric brain arrays. Magn Reson Med 66:1777–1787. 10.1002/mrm.22961 21656548 PMC3218247

[B69] Kelly DJ, Quinn PC, Slater AM, Lee K, Gibson A, Smith M, Ge L, Pascalis O (2005) Three-month-olds, but not newborns, prefer own-race faces. Dev Sci 8:F31–F36. 10.1111/j.1467-7687.2005.0434a.x 16246233 PMC2566511

[B70] Khuvis S, Yeagle EM, Norman Y, Grossman S, Malach R, Mehta AD (2021) Face-selective units in human ventral temporal cortex reactivate during free recall. J Neurosci 41:3386–3399. 10.1523/JNEUROSCI.2918-19.2020 33431634 PMC8051680

[B71] Kinney HC, Ann Brody B, Kloman AS, Gilles FH (1988) Sequence of central nervous system myelination in human infancy. II. Patterns of myelination in autopsied infants. J Neuropathol Exp Neurol 47:217–234. 10.1097/00005072-198805000-00003 3367155

[B72] Kinzler KD, Dupoux E, Spelke ES (2007) The native language of social cognition. Proc Natl Acad Sci U S A 104:12577–12580. 10.1073/pnas.0705345104 17640881 PMC1941511

[B73] Kiorpes L (2015) Visual development in primates: neural mechanisms and critical periods. Dev Neurobiol 75:1080–1090. 10.1002/dneu.22276 25649764 PMC4523497

[B74] Kosakowski HL, Cohen MA, Takahashi A, Keil B, Kanwisher N, Saxe R (2022a) Selective responses to faces, scenes, and bodies in the ventral visual pathway of infants. Curr Biol 32:265–274.e5. 10.1016/j.cub.2021.10.064 34784506 PMC8792213

[B75] Kosakowski HL, Kanwisher N, Saxe R (2022b) Face-preferring regions in FFA, STS, and MPFC have different functions. In: Proceedings of the 44th Annual Conference of the Cognitive Science Society, Presented at the Cognitive Science Society Toronto, Canada.

[B76] Kostović I, Sedmak G, Judaš M (2019) Neural histology and neurogenesis of the human fetal and infant brain. Neuroimage 188:743–773. 10.1016/j.neuroimage.2018.12.043 30594683

[B77] Kourtzi Z, Augath M, Logothetis NK, Movshon JA, Kiorpes L (2006) Development of visually evoked cortical activity in infant macaque monkeys studied longitudinally with fMRI. Magn Reson Imaging 24:359–366. 10.1016/j.mri.2005.12.025 16677941

[B78] Krol KM, Grossmann T (2020) Impression formation in the human infant brain. Cereb Cortex Commun 1:tgaa070. 10.1093/texcom/tgaa070 33134930 PMC7592636

[B79] Kubota E, Grill-Spector K, Nordt M (2024) Rethinking cortical recycling in ventral temporal cortex. Trends Cogn Sci 28:8–17. 10.1016/j.tics.2023.09.006 37858388 PMC10841108

[B80] LaBar KS (2003) Dynamic perception of facial affect and identity in the human brain. Cereb Cortex 13:1023–1033. 10.1093/cercor/13.10.1023 12967919

[B81] Li G, Nie J, Wang L, Shi F, Lin W, Gilmore JH, Shen D (2013) Mapping region-specific longitudinal cortical surface expansion from birth to 2 years of age. Cereb Cortex 23:2724–2733. 10.1093/cercor/bhs265 22923087 PMC3792744

[B82] Lisboa IC, Miguel H, Sampaio A, Mouta S, Santos JA, Pereira AF (2020a) Right STS responses to biological motion in infancy – an fNIRS study using point-light walkers. Neuropsychologia 149:107668. 10.1016/j.neuropsychologia.2020.107668 33137357

[B83] Lisboa IC, Queirós S, Miguel H, Sampaio A, Santos JA, Pereira AF (2020b) Infants’ cortical processing of biological motion configuration – a fNIRS study. Infant Behav Dev 60:101450. 10.1016/j.infbeh.2020.101450 32417706

[B84] Livingstone MS, Vincent JL, Arcaro MJ, Srihasam K, Schade PF, Savage T (2017) Development of the macaque face-patch system. Nat Commun 8:14897. 10.1038/ncomms14897 28361890 PMC5381009

[B85] Lloyd-Fox S, et al. (2017) Cortical specialisation to social stimuli from the first days to the second year of life: a rural Gambian cohort. Dev Cogn Neurosci 25:92–104. 10.1016/j.dcn.2016.11.005 28017265 PMC5485636

[B86] Lloyd-Fox S, Blasi A, Everdell N, Elwell CE, Johnson MH (2011) Selective cortical mapping of biological motion processing in young infants. J Cogn Neurosci 23:2521–2532. 10.1162/jocn.2010.21598 20954934

[B87] Lloyd-Fox S, Blasi A, Volein A, Everdell N, Elwell CE, Johnson MH (2009) Social perception in infancy: a near infrared spectroscopy study. Child Dev 80:986–999. 10.1111/j.1467-8624.2009.01312.x 19630889

[B88] Lloyd-Fox S, Papademetriou M, Darboe MK, Everdell NL, Wegmuller R, Prentice AM, Moore SE, Elwell CE (2014) Functional near infrared spectroscopy (fNIRS) to assess cognitive function in infants in rural Africa. Sci Rep 4:4740. 10.1038/srep04740 24751935 PMC5381189

[B89] Lloyd-Fox S, Széplaki-Köllo˝d B, Yin J, Csibra G (2015) Are you talking to me? Neural activations in 6-month-old infants in response to being addressed during natural interactions. Cortex 70:35–48. 10.1016/j.cortex.2015.02.005 25891796 PMC4636047

[B90] May L, Byers-Heinlein K, Gervain J, Werker JF (2011) Language and the newborn brain: does prenatal language experience shape the neonate neural response to speech? Front Psychol 2:222. 10.3389/fpsyg.2011.00222 21960980 PMC3177294

[B91] Meng Y, Li G, Lin W, Gilmore JH, Shen D (2014) Spatial distribution and longitudinal development of deep cortical sulcal landmarks in infants. Neuroimage 100:206–218. 10.1016/j.neuroimage.2014.06.004 24945660 PMC4138270

[B92] Miller DJ, et al. (2012) Prolonged myelination in human neocortical evolution. Proc Natl Acad Sci U S A 109:16480–16485. 10.1073/pnas.1117943109 23012402 PMC3478650

[B93] Minagawa-Kawai Y, van der Lely H, Ramus F, Sato Y, Mazuka R, Dupoux E (2011) Optical brain imaging reveals general auditory and language-specific processing in early infant development. Cereb Cortex 21:254–261. 10.1093/cercor/bhq082 20497946 PMC3020578

[B94] Naoi N, Minagawa-Kawai Y, Kobayashi A, Takeuchi K, Nakamura K, Yamamoto J, Kojima S (2012) Cerebral responses to infant-directed speech and the effect of talker familiarity. Neuroimage 59:1735–1744. 10.1016/j.neuroimage.2011.07.093 21867764

[B95] Natu VS, Barnett MA, Hartley J, Gomez J, Stigliani A, Grill-Spector K (2016) Development of neural sensitivity to face identity correlates with perceptual discriminability. J Neurosci 36:10893–10907. 10.1523/JNEUROSCI.1886-16.2016 27798143 PMC5083016

[B96] Nieto-Castañón A, Fedorenko E (2012) Subject-specific functional localizers increase sensitivity and functional resolution of multi-subject analyses. Neuroimage 63:1646–1669. 10.1016/j.neuroimage.2012.06.065 22784644 PMC3477490

[B97] Nordt M, Gomez J, Natu VS, Rezai AA, Finzi D, Kular H, Grill-Spector K (2021) Cortical recycling in high-level visual cortex during childhood development. Nat Hum Behav 5:1686–1697. 10.1038/s41562-021-01141-5 34140657 PMC8678383

[B98] O’Doherty J, Winston J, Critchley H, Perrett D, Burt DM, Dolan RJ (2003) Beauty in a smile: the role of medial orbitofrontal cortex in facial attractiveness. Neuropsychologia 41:147–155. 10.1016/S0028-3932(02)00145-8 12459213

[B99] Parvizi J, Jacques C, Foster BL, Withoft N, Rangarajan V, Weiner KS, Grill-Spector K (2012) Electrical stimulation of human fusiform face-selective regions distorts face perception. J Neurosci 32:14915–14920. 10.1523/JNEUROSCI.2609-12.2012 23100414 PMC3517886

[B100] Pascalis O, De Schonen S, Morton J, Deruelle C, Fabre-Grenet M (1995) Mother’s face recognition by neonates: a replication and an extension. Infant Behav Dev 18:79–85. 10.1016/0163-6383(95)90009-8

[B101] Passarotti AM, Paul BM, Bussiere JR, Buxton RB, Wong EC, Stiles J (2003) The development of face and location processing: an fMRI study. Dev Sci 6:100–117. 10.1111/1467-7687.00259

[B102] Peelen MV, Glaser B, Vuilleumier P, Eliez S (2009) Differential development of selectivity for faces and bodies in the fusiform gyrus: development of selectivity for faces and bodies. Dev Sci 12:F16–F25. 10.1111/j.1467-7687.2009.00916.x 19840035

[B103] Pelphrey K, Lopez J, Morris J (2009) Developmental continuity and change in responses to social and nonsocial categories in human extrastriate visual cortex. Front Hum Neurosci 3:885. 10.3389/neuro.09.025.2009 19826492 PMC2759331

[B104] Pitcher D (2022) When is the earliest neural response to faces in the occipital face area? (preprint). PsyArXiv.

[B105] Pitcher D, Dilks DD, Saxe RR, Triantafyllou C, Kanwisher N (2011) Differential selectivity for dynamic versus static information in face-selective cortical regions. Neuroimage 56:2356–2363. 10.1016/j.neuroimage.2011.03.067 21473921

[B106] Pitcher D, Duchaine B, Walsh V (2014) Combined TMS and fMRI reveal dissociable cortical pathways for dynamic and static face perception. Curr Biol 24:2066–2070. 10.1016/j.cub.2014.07.060 25131678

[B107] Pitcher D, Ianni G, Ungerleider LG (2019) A functional dissociation of face-, body- and scene-selective brain areas based on their response to moving and static stimuli. Sci Rep 9:8242. 10.1038/s41598-019-44663-9 31160680 PMC6546694

[B108] Pitcher D, Walsh V, Yovel G, Duchaine B (2007) TMS evidence for the involvement of the right occipital face area in early face processing. Curr Biol 17:1568–1573. 10.1016/j.cub.2007.07.063 17764942

[B109] Polk TA, Park J, Smith MR, Park DC (2007) Nature versus nurture in ventral visual cortex: a functional magnetic resonance imaging study of twins. J Neurosci 27:13921–13925. 10.1523/JNEUROSCI.4001-07.2007 18094229 PMC6673502

[B110] Porto JA, Bick J, Perdue KL, Richards JE, Nunes ML, Nelson CA (2020) The influence of maternal anxiety and depression symptoms on fNIRS brain responses to emotional faces in 5- and 7-month-old infants. Infant Behav Dev 59:101447. 10.1016/j.infbeh.2020.101447 32305734 PMC7255941

[B111] Powell LJ, Deen B, Saxe R (2018) Using individual functional channels of interest to study cortical development with fNIRS. Dev Sci 21:e12595. 10.1111/desc.12595 28944612

[B112] Rangarajan V, Hermes D, Foster BL, Weiner KS, Jacques C, Grill-Spector K, Parvizi J (2014) Electrical stimulation of the left and right human fusiform gyrus causes different effects in conscious face perception. J Neurosci 34:12828 12836. 10.1523/JNEUROSCI.0527-14.2014 25232118 PMC4166163

[B113] Ratan Murty NA, Teng S, Beeler D, Mynick A, Oliva A, Kanwisher N (2020) Visual experience is not necessary for the development of face-selectivity in the lateral fusiform gyrus. Proc Natl Acad Sci U S A 117:23011–23020. 10.1073/pnas.2004607117 32839334 PMC7502773

[B114] Rossion B (2022) Twenty years of investigation with the case of prosopagnosia PS to understand human face identity recognition. Part II: neural basis. Neuropsychologia 173:108279. 10.1016/j.neuropsychologia.2022.108279 35667496

[B115] Rossion B, Hanseeuw B, Dricot L (2012) Defining face perception areas in the human brain: a large-scale factorial fMRI face localizer analysis. Brain Cogn 79:138–157. 10.1016/j.bandc.2012.01.001 22330606

[B116] Rossion B, Lochy A (2022) Is human face recognition lateralized to the right hemisphere due to neural competition with left-lateralized visual word recognition? A critical review. Brain Struct Funct 227:599–629. 10.1007/s00429-021-02370-0 34731327

[B117] Sai FZ (2005) The role of the mother’s voice in developing mother’s face preference: evidence for intermodal perception at birth. Infant Child Dev 14:29–50. 10.1002/icd.376

[B118] Sanai N, et al. (2011) Corridors of migrating neurons in the human brain and their decline during infancy. Nature 478:382–386. 10.1038/nature10487 21964341 PMC3197903

[B119] Sato W, Kochiyama T, Yoshikawa S, Naito E, Matsumura M (2004) Enhanced neural activity in response to dynamic facial expressions of emotion: an fMRI study. Cogn Brain Res 20:81–91. 10.1016/j.cogbrainres.2004.01.008 15130592

[B120] Saxe R, Brett M, Kanwisher N (2006) Divide and conquer: a defense of functional localizers. Neuroimage 30:1088–1096. 10.1016/j.neuroimage.2005.12.062 16635578

[B121] Schalk G, Kapeller C, Guger C, Ogawa H, Hiroshima S, Lafer-Sousa R, Saygin ZM, Kamada K, Kanwisher N (2017) Facephenes and rainbows: causal evidence for functional and anatomical specificity of face and color processing in the human brain. Proc Natl Acad Sci U S A 114:12285–12290. 10.1073/pnas.1713447114 29087337 PMC5699078

[B122] Scherf KS, Behrmann M, Humphreys K, Luna B (2007) Visual category-selectivity for faces, places and objects emerges along different developmental trajectories. Dev Sci 10:F15–F30. 10.1111/j.1467-7687.2007.00595.x 17552930

[B123] Schwarz L, Kreifelts B, Wildgruber D, Erb M, Scheffler K, Ethofer T (2019) Properties of face localizer activations and their application in functional magnetic resonance imaging (fMRI) fingerprinting. PLoS One 14:e0214997. 10.1371/journal.pone.0214997 31013276 PMC6478291

[B124] Scott LS, Arcaro MJ (2023) A domain-relevant framework for the development of face processing. Nat Rev Psychol 2:183–195. 10.1038/s44159-023-00152-5

[B125] Tau GZ, Peterson BS (2010) Normal development of brain circuits. Neuropsychopharmacology 35:147–168. 10.1038/npp.2009.115 19794405 PMC3055433

[B126] Tian X, Hao X, Song Y, Liu J (2021) Homogenization of face neural representation during development. Dev Cogn Neurosci 52:101040. 10.1016/j.dcn.2021.101040 34837875 PMC8637318

[B127] Turati C, Montirosso R, Brenna V, Ferrara V, Borgatti R (2011) A smile enhances 3-month-olds’ recognition of an individual face. Infancy 16:306–317. 10.1111/j.1532-7078.2010.00047.x 32693495

[B128] Tzourio-Mazoyer N, De Schonen S, Crivello F, Reutter B, Aujard Y, Mazoyer B (2002) Neural correlates of woman face processing by 2-month-old infants. Neuroimage 15:454–461. 10.1006/nimg.2001.0979 11798279

[B129] Uchida-Ota M, Arimitsu T, Tsuzuki D, Dan I, Ikeda K, Takahashi T, Minagawa Y (2019) Maternal speech shapes the cerebral frontotemporal network in neonates: a hemodynamic functional connectivity study. Dev Cogn Neurosci 39:100701. 10.1016/j.dcn.2019.100701 31513977 PMC6969365

[B130] Uhlhaas PJ, Roux F, Rodriguez E, Rotarska-Jagiela A, Singer W (2010) Neural synchrony and the development of cortical networks. Trends Cogn Sci 14:72–80. 10.1016/j.tics.2009.12.002 20080054

[B131] Urakawa S, Takamoto K, Ishikawa A, Ono T, Nishijo H (2015) Selective medial prefrontal cortex responses during live mutual gaze interactions in human infants: an fNIRS study. Brain Topogr 28:691–701. 10.1007/s10548-014-0414-2 25367848

[B132] Van Den Hurk J, Van Baelen M, Op De Beeck HP (2017) Development of visual category selectivity in ventral visual cortex does not require visual experience. Proc Natl Acad Sci U S A 114:E4501–E4510. 10.1073/pnas.1612862114 28507127 PMC5465914

[B133] Vasung L, Abaci Turk E, Ferradal SL, Sutin J, Stout JN, Ahtam B, Lin PY, Grant PE (2019) Exploring early human brain development with structural and physiological neuroimaging. Neuroimage 187:226–254. 10.1016/j.neuroimage.2018.07.041 30041061 PMC6537870

[B134] Vouloumanos A, Hauser MD, Werker JF, Martin A (2010) The tuning of human neonates’ preference for speech. Child Dev 81:517–527. 10.1111/j.1467-8624.2009.01412.x 20438457

[B135] Walbrin J, Mihai I, Landsiedel J, Koldewyn K (2020) Developmental changes in visual responses to social interactions. Dev Cogn Neurosci 42:100774. 10.1016/j.dcn.2020.100774 32452460 PMC7075793

[B136] Weiner KS, et al. (2016) The face-processing network is resilient to focal resection of human visual cortex. J Neurosci 36:8425–8440. 10.1523/JNEUROSCI.4509-15.2016 27511014 PMC4978802

[B137] Werchan DM, Collins AGE, Frank MJ, Amso D (2016) Role of prefrontal cortex in learning and generalizing hierarchical rules in 8-month-old infants. J Neurosci 36:10314–10322. 10.1523/JNEUROSCI.1351-16.2016 27707968 PMC5050327

[B138] Xu M, Hoshino E, Yatabe K, Matsuda S, Sato H, Maki A, Yoshimura M, Minagawa Y (2017) Prefrontal function engaging in external-focused attention in 5- to 6-month-old infants: a suggestion for default mode network. Front Hum Neurosci 10), 10.3389/fnhum.2016.00676 28119586 PMC5222871

[B139] Yates TS, Ellis CT, Turk-Browne NB (2021) The promise of awake behaving infant fMRI as a deep measure of cognition. Curr Opin Behav Sci 40:5 11. 10.1016/j.cobeha.2020.11.007

[B140] Yates TS, Ellis CT, Turk-Browne NB (2023) Face processing in the infant brain after pandemic lockdown. Dev Psychobiol 65. 10.1002/dev.22346 36567649 PMC9877889

[B141] Yovel G, Kanwisher N (2005) The neural basis of the behavioral face-inversion effect. Curr Biol 15:2256–2262. 10.1016/j.cub.2005.10.072 16360687

[B142] Yovel G, Tambini A, Brandman T (2008) The asymmetry of the fusiform face area is a stable individual characteristic that underlies the left-visual-field superiority for faces. Neuropsychologia 46:3061–3068. 10.1016/j.neuropsychologia.2008.06.017 18639566

[B143] Zapp J, Schmitter S, Schad LR (2012) Sinusoidal echo-planar imaging with parallel acquisition technique for reduced acoustic noise in auditory fMRI. J Magn Reson Imaging 36:581–588. 10.1002/jmri.23699 22585371

[B144] Zhen Z, Yang Z, Huang L, Kong X, Wang X, Dang X, Huang Y, Song Y, Liu J (2015) Quantifying interindividual variability and asymmetry of face-selective regions: a probabilistic functional atlas. Neuroimage 113:13–25. 10.1016/j.neuroimage.2015.03.010 25772668

