# Supplementary figures and images for: Cortical Face-Selective Responses Emerge Early in Human Infancy
Source: eNeuro. 2024 Jul 16;11(7):ENEURO.0117-24.2024. doi: 10.1523/ENEURO.0117-24.2024 (PMC11258539; doi:10.1523/ENEURO.0117-24.2024)

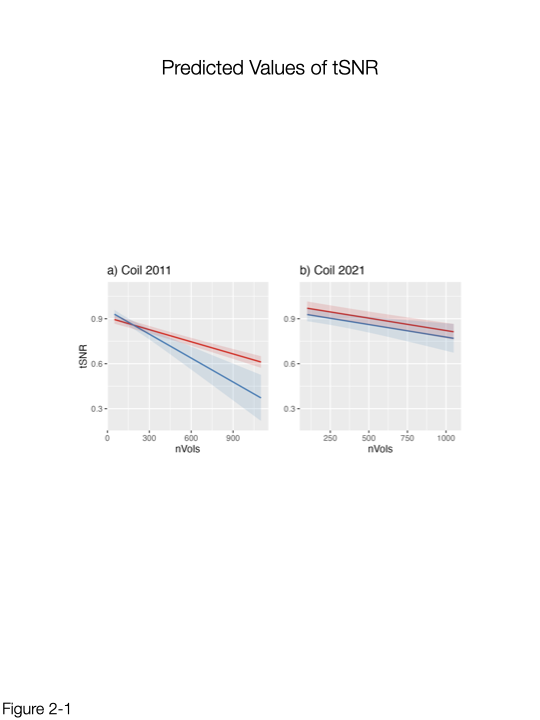

Supplement: Figure 2-1 — Analyses of temporal signal-to-noise ratio (tSNR) in awake infant fMRI data. Plots show estimated effects of run length, run type (i.e., subruns (red) vs concatenated (blue)) on tSNR in (a) Coil 2011 and (b) Coil 2021 data. In Coil 2011 data (a), the tSNR decreases as a function of run length but the decrease is greater when the subruns are concatenated. In Coil 2021 data (b) the tSNR decreases as function of run length but this effect is not modulated by concatenating the runs. All estimated effects are plotted using plot_model from sjPlot package in R. Download Figure 2-1, TIF file. [file eneuro-11-ENEURO.0117-24.2024-s001.tif]

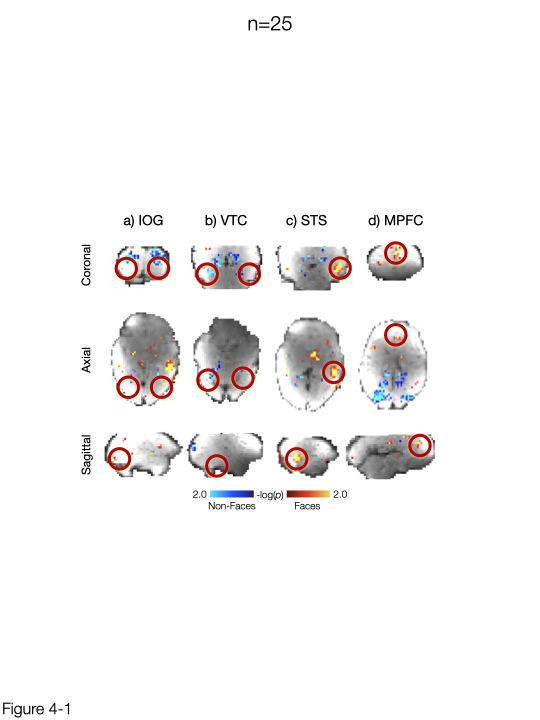

Supplement: Figure 4-1 — Group face responses in infant cerebral cortex. Whole brain group random effects analysis of Coil 2011 at a lenient threshold (p < 0.05) revealed face activations (faces > non-faces) in (c) superior temporal sulcus (STS), and (d) medial prefrontal cortex (MPFC). Face activations were not observed in the group in (a) inferior occipital gyrus (IOG), the approximate location of OFA in adults or (b) ventral temporal cortex (VTC), the approximate location of FFA in adults. Hot colors indicate face activations, cool colors indicate average response to non-faces. Activation clusters did not survive correction for multiple comparisons. Activations for each region are shown on infant template BOLD image in coronal (top row), axial (middle row) and sagittal (bottom row) views and highlighted with a red circle. Results for Coil 2021 data are visualized in Figure 4. Download Figure 4-1, TIF file. [file eneuro-11-ENEURO.0117-24.2024-s002.tif]

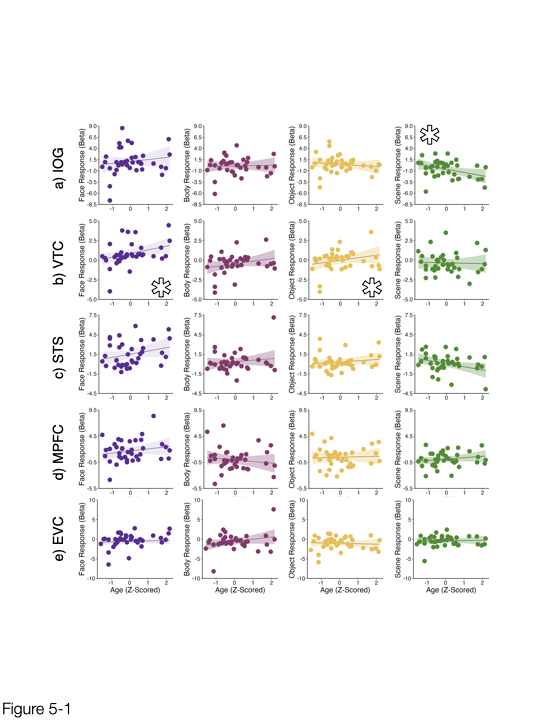

Supplement: Figure 5-1 — Effect of age on the response magnitude for each condition in each fROI. Scatter plots show magnitudes for each condition from fROI analyses collapsed across Coil 2011 and Coil 2021 datasets as a function of age. ROIs include (a) inferior occipital gyrus (IOG), the approximate location of OFA, (b) ventral temporal cortex (VTC), the approximate location of FFA, (c) superior temporal sulcus (STS), (d) medial prefrontal cortex (MPFC), and (e) early visual cortex (EVC). Face betas are plotted in purple, body betas are plotted in pink, object betas are plots in yellow, and scene betas are plotted in green. Age is z-scored. Symbols indicate statistics from linear mixed effects models: p < 0.05. Additional statistics reported in Table 3. Download Figure 5-1, TIF file. [file eneuro-11-ENEURO.0117-24.2024-s003.tif]

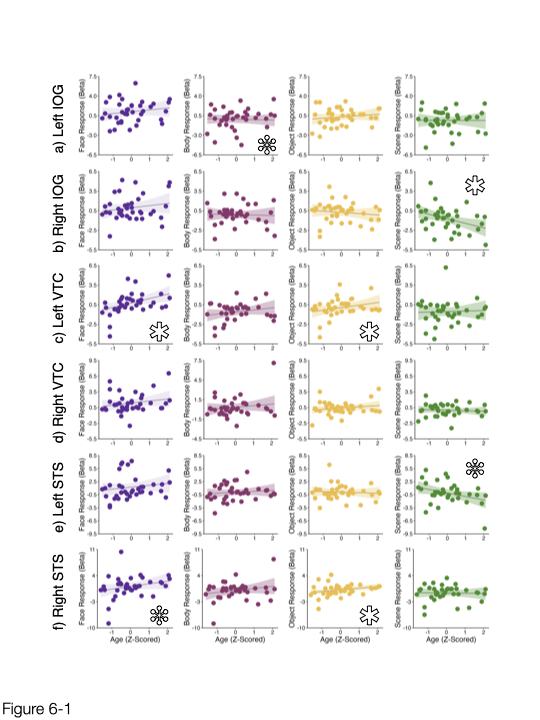

Supplement: Figure 6-1 — Effect of age on the response magnitude for each condition in each fROI. Scatter plots show magnitudes for each condition from fROI analyses collapsed across Coil 2011 and Coil 2021 datasets as a function of age. ROIs include (a, b) left and right inferior occipital gyrus (IOG), the approximate location of OFA, (c, d) left and right ventral temporal cortex (VTC), the approximate location of FFA, and (e, f) superior temporal sulcus (STS). Age is z-scored. Face betas are plotted in purple, body betas are plotted in pink, object betas are plots in yellow, and scene betas are plotted in green. Symbols indicate statistics from linear mixed effects models: p < 0.1; p < 0.05; **p < 0.01. Additional statistics reported in Table 4-2. Download Figure 6-1, TIF file. [file eneuro-11-ENEURO.0117-24.2024-s004.tif]
