# Supplementary material for: Cortical Face-Selective Responses Emerge Early in Human Infancy
Source: eNeuro. 2024 Jul 16;11(7):ENEURO.0117-24.2024. doi: 10.1523/ENEURO.0117-24.2024 (PMC11258539; doi:10.1523/ENEURO.0117-24.2024)
Supplement: Table 1-1 — Face selectivity in functional regions of interest with condition weights. Parameter estimates from linear mixed effects models with beta values for each condition as predictors. Indicator-coded vectors used to test if body, object, and scene responses are each significantly less than the response to faces. Sex and z-scored age were coded as fixed effects and subject was coded as a random effect. Standard error is indicated in paratheses; p < 0.05 is indicated in bold; p < 0.10 is indicated in italics. A negative number in bold indicates a significantly lower response to that condition to faces. The intercept indicates the magnitude of the face response relative to baseline. Statistical models without weights are reported in Table 1. Download Table 1-1, DOC file. [file eneuro-11-ENEURO.0117-24.2024-s005.doc]

| **fROI** | **Intercept** | **Bodies** | **Objects** | **Scenes** | **Age** | **Motion** | **Coil** |
| --- | --- | --- | --- | --- | --- | --- | --- |
| **All Infants** | | | | | | | |
| IOG | **1.41**  **(0.49)** | **-1.43**  **(0.41)** | **-0.85**  **(0.38)** | **-2.19**  **(0.40)** | -0.39  (0.25) | **-0.66**  **(0.24)** | -0.01  (0.58) |
| VTC | **0.66**  **(0.25)** | **-1.27**  **(0.27)** | **-0.70**  **(0.25)** | **-1.30**  **(0.26)** | -0.09  (0.13) | **0.36**  **(0.13)** | 0.42  (0.28) |
| STS | **1.76**  **(0.29)** | **-1.08**  **(0.24)** | **-1.18**  **(0.22)** | **-1.14**  **(0.23)** | -0.00  (0.15) | -0.04  (0.14) | *-0.67*  *(0.35)* |
| MPFC | **1.99 (0.37)** | **-1.57**  **(0.32)** | **-1.29**  **(0.29)** | **-1.39**  **(0.31)** | -0.08  (0.19) | 0.10  (0.18) | -0.63  (0.44) |
| EVC | *-0.47*  *(0.33)* | *-0.49*  *(0.31)* | **-1.01**  **(0.28)** | -0.28  (0.30) | -0.27  (0.17) | 0.19  (0.16) | *0.64*  *(0.38)* |
| **Youngest Infants** | | | | | | | |
| IOG | 0.76  (0.84) | **-1.18**  **(0.58)** | -0.60  (0.55) | **-1.41**  **(0.56)** | -0.27  (0.54) | 0.57  (0.54) | 0.82  (1.03) |
| VTC | 0.16  (0.37) | **-1.16**  **(0.33)** | **-0.62**  **(0.31)** | **-0.87**  **(0.32)** | -0.37  (0.23) | 0.23  (0.22) | 0.49  (0.43) |
| STS | **1.35**  **(0.43)** | **-1.03**  **(0.32)** | **-1.02**  **(0.30)** | **-0.93**  **(0.30)** | 0.21  (0.27) | -0.38  (0.27) | -0.37  (0.52) |
| MPFC | **1.32**  **(0.65)** | **-1.14**  **(0.40)** | **-0.70**  **(0.38)** | **-1.04**  **(0.39)** | 0.05  (0.42) | -0.19  (0.42) | -0.07  (0.81) |
| EVC | *-0.81*  *(0.55)* | *-0.48*  *(0.31)* | **-0.65**  **(0.29)** | -0.28  (0.30) | -0.39  (0.36) | 0.55  (0.36) | 0.83  (0.69) |
| **Oldest Infants** | | | | | | | |
| IOG | **2.18**  **(0.49)** | **-1.63**  **(0.56)** | **-1.09**  **(0.51)** | **-2.99**  **(0.55)** | -0.20  (0.29) | **-0.61**  **(0.26)** | *-0.97*  *(0.55)* |
| VTC | **1.25**  **(0.38)** | **-1.41**  **(0.40)** | **-0.81**  **(0.36)** | **-1.79**  **(0.39)** | 0.03  (0.23) | 0.04  (0.21) | 0.14  (0.44) |
| STS | **1.91**  **(0.36)** | **-1.11**  **(0.36)** | **-1.33**  **(0.32)** | **-1.35**  **(0.35)** | *0.39*  *(0.22)* | 0.16  (0.20) | -0.41  (0.43) |
| MPFC | **2.51**  **(0.52)** | **-2.01**  **(0.48)** | **-1.85**  **(0.43)** | **-1.73**  **(0.47)** | 0.15  (0.33) | 0.22  (0.30) | -0.82  (0.62) |
| EVC | -0.11  (0.40) | -0.48  (0.54) | **-1.36**  **(0.48)** | -0.23  (0.53) | -0.12  (0.21) | 0.23  (0.20) | 0.34  (0.40) |
