# Supplementary material for: Cortical Face-Selective Responses Emerge Early in Human Infancy
Source: eNeuro. 2024 Jul 16;11(7):ENEURO.0117-24.2024. doi: 10.1523/ENEURO.0117-24.2024 (PMC11258539; doi:10.1523/ENEURO.0117-24.2024)
Supplement: Table 2-1 — Interaction effects of age and fROI with condition weights. All results from linear mixed effects models converted to ANOVA table with R function anova; p < 0.05 is indicated in bold, p < 0.10 is indicated in italics. The same models without weights reported in Table 2. Models testing for interaction between age and condition are in each hemisphere are in Table 2-2 (without weights) and Table 2-3 (with weights). Download Table 2-1, DOC file. [file eneuro-11-ENEURO.0117-24.2024-s006.doc]

| **Variable** | **Sum Sq.** | **Num. DF** | **Den. DF** | **F** | **P** |
| --- | --- | --- | --- | --- | --- |
| **IOG** |  |  |  |  |  |
| Condition | **0.46** | **3** | **100.41** | **11.47** | **0.000002** |
| Z-Scored Age | **0.11** | **1** | **89.67** | **8.52** | **0.004** |
| Z-Scored Motion | 0.03 | 1 | 93.90 | 2.53 | 0.11 |
| Coil | 0.00 | 1 | 38.77 | 0.00 | 0.97 |
| Condition * Age | **0.14** | **3** | **100.49** | **3.38** | **0.02** |
| **VTC** |  |  |  |  |  |
| Condition | **0.21** | **3** | **105.99** | **11.02** | **0.000002** |
| Z-Scored Age | **0.05** | **1** | **61.03** | **7.52** | **0.008** |
| Z-Scored Motion | 0.00 | 1 | 73.14 | 0.40 | 0.53 |
| Coil | 0.01 | 1 | 34.58 | 2.31 | 0.14 |
| Condition * Age | 0.03 | 3 | 105.94 | 1.49 | 0.22 |
| **STS** |  |  |  |  |  |
| Condition | **0.19** | **3** | **93.77** | **13.22** | **0.0000003** |
| Z-Scored Age | 0.00 | 1 | 81.37 | 0.09 | 0.77 |
| Z-Scored Motion | 0.00 | 1 | 86.02 | 0.00 | 0.95 |
| Coil | *0.02* | *1* | *32.46* | *3.72* | *0.06* |
| Condition * Age | *0.03* | *3* | *93.86* | *2.20* | *0.09* |
| **MPFC** |  |  |  |  |  |
| Condition | **0.29** | **3** | **105.80** | **11.63** | **0.000001** |
| Z-Scored Age | 0.00 | 1 | 92.07 | 0.17 | 0.68 |
| Z-Scored Motion | 0.00 | 1 | 96.73 | 0.21 | 0.65 |
| Coil | 0.02 | 1 | 43.49 | 2.07 | 0.16 |
| Condition * Age | 0.03 | 3 | 105.87 | 1.26 | 0.29 |
| **EVC** |  |  |  |  |  |
| Condition | **0.10** | **3** | **101.65** | **4.29** | **0.007** |
| Z-Scored Age | 0.01 | 1 | 75.07 | 1.27 | 0.26 |
| Z-Scored Motion | *0.02* | *1* | *82.33* | *2.78* | *0.10* |
| Coil | 0.02 | 1 | 35.61 | 2.80 | 0.10 |
| Condition * Age | **0.06** | **3** | **101.69** | **2.71** | **0.05** |
| **IOG & VTC** |  |  |  |  |  |
| fROI | **0.06** | **1** | **242.74** | **5.24** | **0.02** |
| Condition | **0.63** | **3** | **243.47** | **19.12** | **0.00000000004** |
| Z-Scored Age | 0.01 | 1 | 106.01 | 0.64 | 0.43 |
| Z-Scored Motion | 0.01 | 1 | 111.91 | 1.29 | 0.26 |
| Coil | 0.01 | 1 | 42.30 | 0.75 | 0.39 |
| fROI * Condition | 0.05 | 3 | 242.74 | 1.39 | 0.25 |
| fROI * Age | **0.09** | **1** | **242.74** | **8.50** | **0.004** |
| Condition * Age | **0.14** | **3** | **243.67** | **4.36** | **0.005** |
| fROI * Condition * Age | 0.02 | 3 | 242.74 | 0.64 | 0.59 |
| **EVC & IOG** |  |  |  |  |  |
| fROI | **0.35** | **1** | **242.29** | **32.44** | **0.00000004** |
| Condition | **0.33** | **3** | **242.78** | **10.01** | **0.000003** |
| Z-Scored Age | **0.04** | **1** | **137.02** | **3.95** | **0.048** |
| Z-Scored Motion | **0.04** | **1** | **138.72** | **4.09** | **0.045** |
| Coil | 0.01 | 1 | 49.80 | 1.06 | 0.31 |
| fROI * Condition | **0.26** | **3** | **242.29** | **7.83** | **0.00005** |
| fROI * Age | **0.07** | **1** | **242.29** | **6.89** | **0.01** |
| Condition * Age | **0.14** | **3** | **242.29** | **4.43** | **0.005** |
| fROI * Condition * Age | 0.05 | 3 | 242.29 | 1.65 | 0.18 |
| **EVC & VTC** |  |  |  |  |  |
| fROI | **0.13** | **1** | **239.80** | **16.86** | **0.00006** |
| Condition | **0.21** | **3** | **240.80** | **9.22** | **0.000009** |
| Z-Scored Age | **0.05** | **1** | **84.96** | **6.55** | **0.01** |
| Z-Scored Motion | 0.01 | 1 | 92.80 | 1.31 | 0.26 |
| Coil | **0.04** | **1** | **36.02** | **4.84** | **0.03** |
| fROI * Condition | **0.10** | **3** | **239.80** | **4.61** | **0.004** |
| fROI * Age | 0.00 | 1 | 239.80 | 0.14 | 0.71 |
| Condition * Age | **0.06** | **3** | **239.80** | **2.87** | **0.04** |
| fROI * Condition * Age | 0.02 | 3 | 239.80 | 1.05 | 0.37 |
| **EVC & STS** |  |  |  |  |  |
| fROI | **0.39** | **1** | **243.33** | **46.02** | **0.00000000009** |
| Condition | **0.25** | **3** | **244.65** | **9.76** | **0.000004** |
| Z-Scored Age | 0.01 | 1 | 71.46 | 1.12 | 0.29 |
| Z-Scored Motion | 0.00 | 1 | 82.90 | 0.33 | 0.57 |
| Coil | 0.00 | 1 | 35.44 | 0.00 | 0.96 |
| fROI * Condition | 0.05 | 3 | 243.33 | 2.07 | 0.11 |
| fROI * Age | 0.00 | 1 | 243.33 | 0.03 | 0.87 |
| Condition * Age | **0.07** | **3** | **244.65** | **2.84** | **0.04** |
| fROI * Condition * Age | 0.02 | 3 | 243.33 | 0.86 | 0.46 |
| **EVC & MPFC** |  |  |  |  |  |
| fROI | **0.41** | **1** | **241.10** | **35.01** | **0.00000001** |
| Condition | **0.31** | **3** | **242.63** | **8.89** | **0.00001** |
| Z-Scored Age | 0.02 | 1 | 63.55 | 1.34 | 0.25 |
| Z-Scored Motion | 0.02 | 1 | 75.41 | 1.52 | 0.22 |
| Coil | 0.00 | 1 | 32.36 | 0.00 | 0.997 |
| fROI * Condition | **0.10** | **3** | **241.10** | **2.96** | **0.03** |
| fROI * Age | 0.00 | 1 | 241.10 | 0.07 | 0.79 |
| Condition * Age | 0.07 | 3 | 242.59 | 1.91 | 0.13 |
| fROI * Condition * Age | 0.03 | 3 | 241.10 | 0.78 | 0.50 |
