# Supplementary material for: Cortical Face-Selective Responses Emerge Early in Human Infancy
Source: eNeuro. 2024 Jul 16;11(7):ENEURO.0117-24.2024. doi: 10.1523/ENEURO.0117-24.2024 (PMC11258539; doi:10.1523/ENEURO.0117-24.2024)
Supplement: Table 2-2 — Interaction effects of age in each fROI for each hemisphere. All results from linear mixed effects models converted to ANOVA table with R function anova; p < 0.05 is indicated in bold, p < 0.10 is indicated in italics. Models with weights are in Table 4-4. Download Table 2-2, DOC file. [file eneuro-11-ENEURO.0117-24.2024-s007.doc]

| **Variable** | **Sum Sq.** | **Num. DF** | **Den. DF** | **F** | **P** |
| --- | --- | --- | --- | --- | --- |
| **Left IOG** |  |  |  |  |  |
| Condition | **49.24** | **3** | **105.01** | **9.18** | **0.00002** |
| Z-Scored Age | 1.76 | 1 | 88.20 | 0.98 | 0.32 |
| Z-Scored Motion | 1.40 | 1 | 86.05 | 0.79 | 0.38 |
| Coil | 5.83 | 2 | 35.74 | 1.63 | 0.21 |
| Condition * Age | 2.75 | 3 | 105.01 | 0.51 | 0.67 |
| **Right IOG** |  |  |  |  |  |
| Condition | **57.64** | **3** | **106.26** | **8.89** | **0.00003** |
| Z-Scored Age | *6.04* | *1* | *64.71* | *2.79* | *0.10* |
| Z-Scored Motion | *6.46* | *1* | *63.82* | *2.99* | *0.09* |
| Coil | 0.33 | 2 | 35.22 | 0.08 | 0.93 |
| Condition * Age | **26.46** | **3** | **106.26** | **4.08** | **0.009** |
| **Left VTC** |  |  |  |  |  |
| Condition | **43.06** | **3** | **106.83** | **7.80** | **0.00009** |
| Z-Scored Age | **13.22** | **1** | **63.34** | **7.19** | **0.009** |
| Z-Scored Motion | *6.72* | *1* | *62.53* | *3.65* | *0.06* |
| Coil | 5.93 | 2 | 35.71 | 1.61 | 0.21 |
| Condition * Age | 11.77 | 3 | 106.83 | 2.13 | 0.10 |
| **Right VTC** |  |  |  |  |  |
| Condition | **38.59** | **3** | **104.52** | **8.81** | **0.00003** |
| Z-Scored Age | 0.80 | 1 | 84.08 | 0.55 | 0.46 |
| Z-Scored Motion | 1.90 | 1 | 82.11 | 1.30 | 0.26 |
| Coil | 6.88 | 2 | 34.94 | 2.36 | 0.11 |
| Condition * Age | 4.46 | 3 | 104.52 | 1.02 | 0.39 |
| **Left STS** |  |  |  |  |  |
| Condition | **35.66** | **3** | **106.66** | **3.67** | **0.01** |
| Z-Scored Age | 0.00 | 1 | 78.12 | 0.00 | 0.99 |
| Z-Scored Motion | 2.38 | 1 | 76.64 | 0.74 | 0.39 |
| Coil | 1.11 | 2 | 36.60 | 0.17 | 0.84 |
| Condition * Age | **36.50** | **3** | **106.66** | **3.76** | **0.01** |
| **Right STS** |  |  |  |  |  |
| Condition | **55.11** | **3** | **105.31** | **6.76** | **0.0003** |
| Z-Scored Age | 2.05 | 1 | 97.80 | 0.75 | 0.39 |
| Z-Scored Motion | 0.81 | 1 | 95.19 | 0.30 | 0.59 |
| Coil | *14.52* | *2* | *36.89* | *2.67* | *0.08* |
| Condition * Age | 15.73 | 3 | 105.31 | 1.93 | 0.13 |
