# Supplementary material for: Cortical Face-Selective Responses Emerge Early in Human Infancy
Source: eNeuro. 2024 Jul 16;11(7):ENEURO.0117-24.2024. doi: 10.1523/ENEURO.0117-24.2024 (PMC11258539; doi:10.1523/ENEURO.0117-24.2024)
Supplement: Table 2-3 — Interaction effects of age in each fROI for each hemisphere with condition weights. All results from linear mixed effects models converted to ANOVA with R function anova; p < 0.05 is indicated in bold, p < 0.10 is indicated in italics. Models without weights are in Table 4-3. Download Table 2-3, DOC file. [file eneuro-11-ENEURO.0117-24.2024-s008.doc]

| **Variable** | **Sum Sq.** | **Num. DF** | **Den. DF** | **F** | **P** |
| --- | --- | --- | --- | --- | --- |
| **Left IOG** |  |  |  |  |  |
| Condition | **0.22** | **3** | **104.97** | **10.81** | **0.000003** |
| Z-Scored Age | 0.01 | 1 | 101.64 | 1.80 | 0.18 |
| Z-Scored Motion | 0.01 | 1 | 102.64 | 1.33 | 0.25 |
| Coil | 0.03 | 2 | 38.50 | 1.82 | 0.18 |
| Condition * Age | 0.01 | 3 | 105.04 | 0.50 | 0.68 |
| **Right IOG** |  |  |  |  |  |
| Condition | **0.28** | **3** | **106.82** | **9.86** | **0.000009** |
| Z-Scored Age | **0.06** | **1** | **71.13** | **5.92** | **0.02** |
| Z-Scored Motion | 0.02 | 1 | 79.25 | 2.54 | 0.11 |
| Coil | 0.01 | 2 | 38.02 | 0.32 | 0.73 |
| Condition * Age | **0.10** | **3** | **106.77** | **3.61** | **0.02** |
| **Left VTC** |  |  |  |  |  |
| Condition | **0.25** | **3** | **102.95** | **11.26** | **0.000002** |
| Z-Scored Age | **0.07** | **1** | **60.15** | **8.94** | **0.004** |
| Z-Scored Motion | 0.02 | 1 | 70.16 | 2.28 | 0.14 |
| Coil | 0.03 | 2 | 33.28 | 1.79 | 0.18 |
| Condition * Age | 0.05 | 3 | 102.86 | 2.13 | 0.10 |
| **Right VTC** |  |  |  |  |  |
| Condition | **0.19** | **3** | **99.54** | **10.22** | **0.000006** |
| Z-Scored Age | 0.00 | 1 | 84.23 | 0.44 | 0.51 |
| Z-Scored Motion | 0.06 | 1 | 87.21 | 0.95 | 0.33 |
| Coil | *0.04* | *2* | *32.23* | *2.83* | *0.07* |
| Condition * Age | 0.03 | 3 | 99.59 | 1.85 | 0.14 |
| **Left STS** |  |  |  |  |  |
| Condition | **0.32** | **3** | **102.24** | **6.40** | **0.0005** |
| Z-Scored Age | 0.00 | 1 | 66.79 | 0.08 | 0.78 |
| Z-Scored Motion | 0.01 | 1 | 74.41 | 0.43 | 0.51 |
| Coil | 0.02 | 2 | 33.33 | 0.54 | 0.59 |
| Condition * Age | *0.13* | *3* | *102.19* | *2.54* | *0.06* |
| **Right STS** |  |  |  |  |  |
| Condition | **0.35** | **3** | **106.91** | **8.84** | **0.00003** |
| Z-Scored Age | 0.01 | 1 | 104.23 | 0.48 | 0.49 |
| Z-Scored Motion | 0.00 | 1 | 105.12 | 0.26 | 0.61 |
| Coil | 0.05 | 2 | 40.83 | 1.72 | 0.19 |
| Condition * Age | 0.06 | 3 | 106.98 | 1.51 | 0.22 |
