# Supplementary material for: Cortical Face-Selective Responses Emerge Early in Human Infancy
Source: eNeuro. 2024 Jul 16;11(7):ENEURO.0117-24.2024. doi: 10.1523/ENEURO.0117-24.2024 (PMC11258539; doi:10.1523/ENEURO.0117-24.2024)
Supplement: Table 3-1 — Effect of age on each condition with condition weights. † Parameters estimated with a linear-mixed effects model in R. Condition response indicated in the left column are the predictors, z-scored age coded as a fixed effect, subject coded as a random effect. Standard error is indicated in paratheses. p < 0.05 is indicated in bold, p < 0.10 is indicated in italics. * Model was singular due to negligible contribution of participant in the random effects term. A linear model without subject as a random effect produces the same results without a singular fit. Statistical models without weights are reported in Table 3. Download Table 3-1, DOC file. [file eneuro-11-ENEURO.0117-24.2024-s009.doc]

| **fROI** | **Intercept^†^** | **Age^†^** | **Motion^†^** | **Coil^†^** |
| --- | --- | --- | --- | --- |
| IOG Face | **2.29**  **(0.73)** | 0.03  (0.48) | -0.64  (0.51) | -1.43  (1.00) |
| IOG Body* | 0.03  (0.46) | -0.06  (0.29) | -0.18  (0.33) | 0.26  (0.60) |
| IOG Object* | **0.90**  **(0.42)** | -0.32  (0.30) | 0.14  (0.32) | -0.19  (0.60) |
| IOG Scene | **-1.22**  **(0.42)** | **-0.95**  **(0.25)** | *-0.49*  *(0.29)* | -0.49  (0.54) |
| VTC Face | **1.00**  **(0.35)** | **0.47**  **(0.21)** | -0.10  (0.22) | -0.24  (0.47) |
| VTC Body* | **-0.73**  **(0.28)** | *0.35*  *(0.18)* | **-0.45**  **(0.20)** | *0.63*  *(0.37)* |
| VTC Object | -0.14  (0.27) | 0.29  (0.19) | -0.20  (0.20) | 0.65  (0.39) |
| VTC Scene | **-0.78**  **(0.37)** | 0.03  (0.22) | 0.06  (0.25) | 0.72  (0.47) |
| STS Face* | **2.18**  **(0.38)** | 0.28  (0.26) | 0.41  (0.28) | **-1.31**  **(0.52)** |
| STS Body* | 0.53  (0.34) | *0.37*  *(0.22)* | **0.58**  **(0.24)** | -0.23  (0.44) |
| STS Object | 0.34  (0.25) | 0.17  (0.18) | 0.30  (0.19) | -0.04  (0.36) |
| STS Scene* | 0.43  (0.28) | -0.16  (0.17) | *0.35*  *(0.20)* | -0.22  (0.35) |
| MPFC Face | **2.27**  **(0.45)** | 0.01  (0.14) | *-0.77*  *(0.15)* | *-1.32*  *(0.47)* |
| MPFC Body | -0.08  (0.52) | -0.14  (0.32) | 0.07  (0.34) | 0.07  (0.69) |
| MPFC Object* | **0.91**  **(0.38)** | 0.09  (0.26) | 0.39  (0.28) | -0.87  (0.54) |
| MPFC Scene | 0.29  (0.29) | 0.25  (0.16) | -0.28  (0.18) | -0.11  (0.39) |
| EVC Face* | 0.03  (0.35) | *0.42*  *(0.24)* | **-0.55**  **(0.26)** | -0.22  (0.48) |
| EVC Body | -0.60  (0.54) | 0.42  (0.35) | 0.31  (0.39) | 0.20  (0.72) |
| EVC Object | **-1.81**  **(0.33)** | -0.07  (0.22) | -0.23  (0.22) | **1.42**  **(0.46)** |
| EVC Scene* | *-0.53*  *(0.30)* | 0.16  (0.18) | *-0.36*  *(0.21)* | 0.35  (0.38) |
