# Supplementary material for: Cortical Face-Selective Responses Emerge Early in Human Infancy
Source: eNeuro. 2024 Jul 16;11(7):ENEURO.0117-24.2024. doi: 10.1523/ENEURO.0117-24.2024 (PMC11258539; doi:10.1523/ENEURO.0117-24.2024)
Supplement: Table 4-1 — Face selectivity in each fROI for each hemisphere. Parameter estimates from linear mixed effects models with beta values for each condition as predictors. Indicator-coded vectors used to test if body, object, and scene responses are each significantly less than the response to faces. Sex and z-scored age were coded as fixed effects and subject was coded as a random effect. Standard error is indicated in paratheses; p < 0.05 is indicated in bold; p < 0.10 is indicated in italics. A negative number in bold indicates a significantly lower response to that condition to faces. The intercept indicates the magnitude of the face response relative to baseline. Models with weights in Table 4-2. Download Table 4-1, DOC file. [file eneuro-11-ENEURO.0117-24.2024-s010.doc]

| **fROI** | **Intercept** | **Bodies** | **Objects** | **Scenes** | **Age** | **Motion** | **Coil** |
| --- | --- | --- | --- | --- | --- | --- | --- |
| **All Infants** | | | | | | | |
| Left IOG | **1.34**  **(0.39)** | **-1.23**  **(0.31)** | **-0.91**  **(0.31)** | **-1.54**  **(0.31)** | -0.17  (0.19) | -0.19  (0.19) | 1.25  (1.07) |
| Right IOG | **0.84**  **(0.35)** | **-1.06**  **(0.36)** | **-0.70**  **(0.36)** | **-1.73**  **(0.36)** | *-0.29*  *(0.17)* | -0.27  (0.17) | 0.21  (0.85) |
| Left VTC | *0.44*  *(0.31)* | **-1.32**  **(0.32)** | **-0.66**  **(0.32)** | **-1.29**  **(0.32)** | *-0.29*  *(0.15)* | **0.41**  **(0.15)** | -0.12  (0.76) |
| Right VTC | **1.82**  **(0.34)** | **-1.06**  **(0.28)** | **-0.96**  **(0.28)** | **-1.36**  **(0.28)** | 0.19  (0.17) | 0.12  (0.17) | -0.77  (0.93) |
| Left STS | **1.33**  **(0.48)** | **-1.08**  **(0.43)** | **-1.06**  **(0.43)** | **-1.23**  **(0.43)** | -0.19  (0.23) | -0.00  (0.24) | 0.05  (1.24) |
| Right STS | 0.33  (0.52) | **-1.02**  **(0.39)** | **-0.93**  **(0.39)** | **-1.72**  **(0.39)** | 0.13  (0.25) | 0.22  (0.25) | 1.23  (1.47) |
