# Supplementary material for: Cortical Face-Selective Responses Emerge Early in Human Infancy
Source: eNeuro. 2024 Jul 16;11(7):ENEURO.0117-24.2024. doi: 10.1523/ENEURO.0117-24.2024 (PMC11258539; doi:10.1523/ENEURO.0117-24.2024)
Supplement: Table 4-2 — Face selectivity in fROI for each hemisphere with condition weights. Parameter estimates from linear mixed effects models with beta values for each condition as predictors. Indicator-coded vectors used to test if body, object, and scene responses are each significantly less than the response to faces. Sex and z-scored age were coded as fixed effects and subject was coded as a random effect. Standard error is indicated in paratheses; p < 0.05 is indicated in bold; p < 0.10 is indicated in italics. A negative number in bold indicates a significantly lower response to that condition to faces. The intercept indicates the magnitude of the face response relative to baseline. Models without weights in Table 4-1. Download Table 4-2, DOC file. [file eneuro-11-ENEURO.0117-24.2024-s011.doc]

| **fROI** | **Intercept** | **Bodies** | **Objects** | **Scenes** | **Age** | **Motion** | **Coil** |
| --- | --- | --- | --- | --- | --- | --- | --- |
| **All Infants** | | | | | | | |
| Left IOG | **1.31 (0.37)** | **-1.18**  **(0.28)** | **-0.95**  **(0.26)** | **-1.44**  **(0.27)** | -0.21  (0.18) | -0.24  (0.17) | 1.58  (1.10) |
| Right IOG | **0.10**  **(0.33)** | **-1.24**  **(0.34)** | **-0.69**  **(0.31)** | **-1.64**  **(0.33)** | -0.27  (0.17) | **-0.37**  **(0.17)** | 0.74  (0.91) |
| Left VTC | **0.57**  **(0.28)** | **-1.36**  **(0.30)** | **-0.72**  **(0.27)** | **-1.46**  **(0.29)** | -0.22  (0.15) | **0.45**  **(0.14)** | -0.19  (0.76) |
| Right VTC | **1.87**  **(0.32)** | **-1.02**  **(0.27)** | **-1.03**  **(0.25)** | **-1.30**  **(0.26)** | 0.16  (0.16) | 0.11  (0.16) | -0.66  (0.92) |
| Left STS | **1.75**  **(0.44)** | **-1.47**  **(0.45)** | **-1.28**  **(0.42)** | **-1.65**  **(0.44)** | -0.15  (0.23) | -0.06  (0.22) | 0.01  (1.23) |
| Right STS | 0.65  (0.51) | **-1.26**  **(0.39)** | **-0.94**  **(0.36)** | **-1.91**  **(0.38)** | 0.12  (0.25) | 0.17  (0.24) | 1.11  (1.52) |
