# Supplementary material for: Cortical Face-Selective Responses Emerge Early in Human Infancy
Source: eNeuro. 2024 Jul 16;11(7):ENEURO.0117-24.2024. doi: 10.1523/ENEURO.0117-24.2024 (PMC11258539; doi:10.1523/ENEURO.0117-24.2024)
Supplement: Table 4-3 — Effect of age and hemisphere on face Selectivity in each fROI with condition weights. All results from linear mixed effects models converted to ANOVA with R function anova; p < 0.05 is indicated in bold, p < 0.10 is indicated in italics. Statistics for models without weights are reported in Table 4. Download Table 4-3, DOC file. [file eneuro-11-ENEURO.0117-24.2024-s012.doc]

| **Variable** | **Sum Sq.** | **Num. DF** | **Den. DF** | **F** | **P** |
| --- | --- | --- | --- | --- | --- |
| **IOG** |  |  |  |  |  |
| Hemisphere | 0.01 | 1 | 243.10 | 0.56 | 0.46 |
| Condition | **0.49** | **3** | **244.07** | **16.79** | **0.0000000006** |
| Z-Scored Age | **0.05** | **1** | **94.26** | **5.61** | **0.02** |
| Z-Scored Motion | 0.03 | 1 | 99.15 | 2.67 | 0.11 |
| Coil | 0.03 | 2 | 35.92 | 1.53 | 0.23 |
| Hemisphere * Condition | 0.01 | 3 | 243.10 | 0.34 | 0.80 |
| Hemisphere * Age | **0.06** | **1** | **243.10** | **6.57** | **0.01** |
| Condition * Age | **0.08** | **3** | **244.15** | **2.89** | **0.04** |
| Hemi * Condition * Age | 0.02 | 3 | 243.10 | 0.84 | 0.47 |
| **VTC** |  |  |  |  |  |
| Hemisphere | **0.11** | **1** | **251.67** | **11.47** | **0.0008** |
| Condition | **0.41** | **3** | **253.69** | **14.31** | **0.00000001** |
| Z-Scored Age | **0.10** | **1** | **62.43** | **10.13** | **0.002** |
| Z-Scored Motion | 0.00 | 1 | 83.28 | 0.10 | 0.75 |
| Coil | 0.02 | 2 | 43.15 | 0.98 | 0.38 |
| Hemisphere * Condition | 0.01 | 3 | 251.67 | 0.46 | 0.71 |
| Hemisphere * Age | 0.00 | 1 | 251.67 | 0.19 | 0.66 |
| Condition * Age | *0.07* | *3* | *253.36* | *2.44* | *0.06* |
| Hemi * Condition * Age | 0.01 | 3 | 251.67 | 0.43 | 0.73 |
| **STS** |  |  |  |  |  |
| Hemisphere | 0.00 | 1 | 245.26 | 0.00 | 1.00 |
| Condition | **0.65** | **3** | **246.01** | **12.34** | **0.0000002** |
| Z-Scored Age | 0.00 | 1 | 111.77 | 0.13 | 0.72 |
| Z-Scored Motion | 0.00 | 1 | 114.77 | 0.12 | 0.73 |
| Coil | 0.01 | 2 | 38.87 | 0.35 | 0.70 |
| Hemisphere * Condition | 0.01 | 3 | 245.26 | 0.28 | 0.84 |
| Hemisphere * Age | 0.01 | 1 | 245.26 | 0.62 | 0.43 |
| Condition * Age | **0.18** | **3** | **246.14** | **3.47** | **0.02** |
| Hemi * Condition * Age | 0.01 | 3 | 245.26 | 0.13 | 0.94 |
