# Supplementary material for: Cortical Face-Selective Responses Emerge Early in Human Infancy
Source: eNeuro. 2024 Jul 16;11(7):ENEURO.0117-24.2024. doi: 10.1523/ENEURO.0117-24.2024 (PMC11258539; doi:10.1523/ENEURO.0117-24.2024)
Supplement: Table 4-4 — Effect of Age on each condition in each hemisphere. † Parameters estimated with a linear-mixed effects model in R. Condition response indicated in the left column are the predictors, z-scored age coded as a fixed effect, subject coded as a random effect. Standard error is indicated in paratheses. p < 0.05 is indicated in bold, p < 0.10 is indicated in italics. * Model was singular due to negligible contribution of participant in the random effects term. A linear model without subject as a random effect produces the same results without a singular fit. No weights included in analyses. Download Table 4-4, DOC file. [file eneuro-11-ENEURO.0117-24.2024-s013.doc]

| **fROI** | **Intercept^†^** | **Age^†^** | **Motion^†^** | **Coil^†^** |
| --- | --- | --- | --- | --- |
| Left IOG  Face | **1.97**  **(0.43)** | 0.14  (0.30) | -0.42  (0.29) | **-1.42**  **(0.59)** |
| Left IOG  Body | 0.25  (0.35) | *-0.29*  *(0.05)* | *0.39*  *(0.05)* | -0.80  (0.17) |
| Left IOG Object* | 0.14  (0.38) | 0.23  (0.26) | -0.14  (0.26) | 0.28  (0.52) |
| Left IOG Scene* | -0.12  (0.46) | -0.05  (0.31) | -0.01  (0.31) | -0.40  (0.63) |
| Right IOG  Face* | 1.30  (0.45) | 0.28  (0.30) | -0.53  (0.30) | -0.65  (0.61) |
| Right IOG  Body* | -0.36  (0.38) | -0.14  (0.26) | -0.34  (0.26) | 0.46  (0.52) |
| Right IOG Object* | -0.02  (0.38) | -0.06  (0.26) | -0.15  (0.25) | 0.48  (0.51) |
| Right IOG Scene | -0.44  (0.30) | **-0.96**  **(0.02)** | **-0.48**  **(0.02)** | *-0.62*  *(0.07)* |
| Left VTC  Face | **0.89**  **(0.37)** | **0.72**  **(0.24)** | -0.24  (0.24) | -0.26  (0.51) |
| Left VTC  Body* | **-0.96**  **(0.27)** | 0.20  (0.19) | **-0.84**  **(0.18)** | *0.64*  *(0.37)* |
| Left VTC  Object* | *-0.60*  *(0.31)* | **0.48**  **(0.21)** | 0.08  (0.21) | **1.19**  **(0.43)** |
| Left VTC  Scene | *-0.86*  *(0.45)* | 0.10  (0.30) | -0.21  (0.29) | 0.58  (0.61) |
| Right VTC  Face | **2.40**  **(0.39)** | 0.27  (0.27) | 0.35  (0.27) | **-1.92**  **(0.54)** |
| Right VTC  Body | 0.16  (0.38) | 0.02  (0.06) | **0.71**  **(0.06)** | 0.17  (0.21) |
| Right VTC  Object | *0.58*  *(0.31)* | 0.20  (0.18) | 0.13  (0.18) | -0.33  (0.41) |
| Right VTC  Scene | 0.16  (0.29) | -0.07  (0.18) | 0.10  (0.18) | -0.34  (0.39) |
| Left STS  Face | **1.92**  **(0.62)** | 0.27  (0.39) | -0.44  (0.39) | -1.35  (0.85) |
| Left STS  Body* | 0.11  (0.45) | 0.30  (0.31) | 0.13  (0.31) | -0.13  (0.62) |
| Left STS  Object* | 0.48  (0.43) | -0.03  (0.30) | 0.11  (0.29) | -0.77  (0.59) |
| Left STS  Scene | -0.47  (0.58) | *-0.70*  *(0.35)* | -0.31  (0.34) | 0.68  (0.77) |
| Right STS  Face | 0.50  (0.70) | *0.81*  *(0.42)* | -0.18  (0.42) | 1.13  (0.94) |
| Right STS  Body* | -0.42  (0.64) | 0.58  (0.44) | -0.12  (0.43) | 0.82  (0.88) |
| Right STS  Object | 0.12  (0.36) | **0.45**  **(0.15)** | 0.03  (0.15) | 0.15  (0.44) |
| Right STS  Scene* | **-1.53**  **(0.46)** | 0.00  (0.32) | -0.39  (0.31) | **1.60**  **(0.64)** |
