# Supplementary material for: Cortical Face-Selective Responses Emerge Early in Human Infancy
Source: eNeuro. 2024 Jul 16;11(7):ENEURO.0117-24.2024. doi: 10.1523/ENEURO.0117-24.2024 (PMC11258539; doi:10.1523/ENEURO.0117-24.2024)
Supplement: Table 4-5 — Effect of age and hemisphere on condition responses for each fROI. All results from linear mixed effects models converted to ANOVA with R function anova; p < 0.05 is indicated in bold, p < 0.10 is indicated in italics. No weights included in analyses. Download Table 4-5, DOC file. [file eneuro-11-ENEURO.0117-24.2024-s014.doc]

| **Variable** | **Sum Sq.** | **Num. DF** | **Den. DF** | **F** | **P** |
| --- | --- | --- | --- | --- | --- |
| **Left & Right IOG** |  |  |  |  |  |
| **Faces** |  |  |  |  |  |
| Hemisphere | 1.2802 | 1 | 33.428 | 0.5182 | 0.47663 |
| Z-Scored Age | 0.5932 | 1 | 48.413 | 0.2401 | 0.62636 |
| Z-Scored Motion | **10.3547** | **1** | **47.793** | **4.1912** | **0.04615** |
| Coil | **11.5160** | **1** | **32.546** | **4.6612** | **0.03832** |
| Hemisphere * Age | 0.1703 | 1 | 33.428 | 0.0689 | 0.79450 |
| **Bodies** |  |  |  |  |  |
| Hemisphere | 0.1507 | 1 | 37.678 | 0.0590 | 0.8094 |
| Z-Scored Age | 0.1373 | 1 | 47.627 | 0.0537 | 0.8177 |
| Z-Scored Motion | 1.9297 | 1 | 46.641 | 0.7550 | 0.3894 |
| Coil | 0.0555 | 1 | 35.511 | 0.0217 | 0.8837 |
| Hemisphere * Age | 4.3587 | 1 | 37.678 | 1.7053 | 0.1995 |
| **Objects** |  |  |  |  |  |
| Hemisphere | 0.05279 | 1 | 35.570 | 0.0289 | 0.8659 |
| Z-Scored Age | 0.00069 | 1 | 35.570 | 0.0004 | 0.9845 |
| Z-Scored Motion | 0.99210 | 1 | 49.288 | 0.5439 | 0.4643 |
| Coil | 1.29183 | 1 | 34.619 | 0.7082 | 0.4058 |
| Hemisphere * Age | 1.75373 | 1 | 35.570 | 0.9615 | 0.3334 |
| **Scenes** |  |  |  |  |  |
| Hemisphere | 3.7284 | 1 | 38.264 | 1.5704 | 0.21775 |
| Z-Scored Age | **10.2582** | **1** | **52.814** | **4.3207** | **0.04253** |
| Z-Scored Motion | 1.3856 | 1 | 52.315 | 0.5836 | 0.44833 |
| Coil | 0.1959 | 1 | 37.607 | 0.0825 | 0.77550 |
| Hemisphere * Age | **11.5100** | **1** | **38.264** | **4.8480** | **0.03378** |
| **Left & Right VTC** |  |  |  |  |  |
| **Faces** |  |  |  |  |  |
| Hemisphere | 1.0193 | 1 | 33.614 | 0.5757 | 0.45329 |
| Z-Scored Age | *6.5363* | *1* | *54.946* | *3.6918* | *0.05987* |
| Z-Scored Motion | 3.9049 | 1 | 54.553 | 2.2055 | 0.14327 |
| Coil | 1.1795 | 1 | 34.638 | 0.6662 | 0.41996 |
| Hemisphere * Age | 3.3390 | 1 | 33.614 | 1.8859 | 0.17875 |
| **Bodies** |  |  |  |  |  |
| Hemisphere | **4.5808** | **1** | **36.990** | **4.2393** | **0.046591** |
| Z-Scored Age | 0.0002 | 1 | 59.075 | 0.0002 | 0.989468 |
| Z-Scored Motion | **10.9554** | **1** | **58.755** | **10.1386** | **0.002323** |
| Coil | 1.9122 | 1 | 38.844 | 1.7696 | 0.191184 |
| Hemisphere * Age | 2.3956 | 1 | 36.990 | 2.2169 | 0.144980 |
| **Objects** |  |  |  |  |  |
| Hemisphere | 0.6766 | 1 | 35.985 | 0.4965 | 0.48558 |
| Z-Scored Age | 1.4417 | 1 | 54.416 | 1.0579 | 0.30824 |
| Z-Scored Motion | 0.0109 | 1 | 54.015 | 0.0080 | 0.92898 |
| Coil | **6.1745** | **1** | **36.343** | **4.5310** | **0.04013** |
| Hemisphere * Age | *4.0557* | *1* | *35.985* | *2.9761* | *0.09308* |
| **Scenes** |  |  |  |  |  |
| Hemisphere | 0.8060 | 1 | 41.465 | 0.3643 | 0.54941 |
| Z-Scored Age | 5.6982 | 1 | 55.325 | 2.5757 | 0.11421 |
| Z-Scored Motion | 3.0172 | 1 | 54.900 | 1.3638 | 0.24792 |
| Coil | 1.8915 | 1 | 40.949 | 0.8550 | 0.36056 |
| Hemisphere * Age | **13.4385** | **1** | **41.465** | **6.0744** | **0.01795** |
| **Left & Right STS** |  |  |  |  |  |
| **Faces** |  |  |  |  |  |
| Hemisphere | 0.6239 | 1 | 34.901 | 0.1833 | 0.6711 |
| Z-Scored Age | 2.2069 | 1 | 52.831 | 0.6485 | 0.4242 |
| Z-Scored Motion | 8.3197 | 1 | 52.388 | 2.4450 | 0.1239 |
| Coil | 9.3355 | 1 | 34.985 | 2.7435 | 0.1066 |
| Hemisphere * Age | 0.2171 | 1 | 34.901 | 0.0638 | 0.8021 |
| **Bodies** |  |  |  |  |  |
| Hemisphere | 0.4687 | 1 | 31.606 | 0.1676 | 0.6850 |
| Z-Scored Age | 0.4867 | 1 | 39.584 | 0.1741 | 0.6788 |
| Z-Scored Motion | 0.7956 | 1 | 38.019 | 0.2846 | 0.5968 |
| Coil | 0.4272 | 1 | 28.656 | 0.1528 | 0.6988 |
| Hemisphere * Age | 3.7227 | 1 | 31.606 | 1.3314 | 0.2572 |
| **Objects** |  |  |  |  |  |
| Hemisphere | 0.55232 | 1 | 36.749 | 0.2570 | 0.6152 |
| Z-Scored Age | 0.30956 | 1 | 51.284 | 0.1441 | 0.7059 |
| Z-Scored Motion | 0.02466 | 1 | 50.734 | 0.0115 | 0.9151 |
| Coil | 0.37033 | 1 | 35.950 | 0.1723 | 0.6805 |
| Hemisphere * Age | 0.22442 | 1 | 36.749 | 0.1044 | 0.7484 |
| **Scenes** |  |  |  |  |  |
| Hemisphere | 8.654 | 1 | 68 | 2.1027 | 0.15164 |
| Z-Scored Age | **43.601** | **1** | **68** | **10.5937** | **0.00177** |
| Z-Scored Motion | 4.965 | 1 | 68 | 1.2064 | 0.27592 |
| Coil | 7.088 | 1 | 68 | 1.7223 | 0.19381 |
| Hemisphere * Age | 0.000 | 1 | 68 | 0.0000 | 0.99476 |
